# Cell Intrinsic Galectin-3 Attenuates Neutrophil ROS-Dependent Killing of *Candida* by Modulating CR3 Downstream Syk Activation

**DOI:** 10.3389/fimmu.2017.00048

**Published:** 2017-02-03

**Authors:** Sheng-Yang Wu, Juin-Hua Huang, Wen-Yu Chen, Yi-Chen Chan, Chun-Hung Lin, Yee-Chun Chen, Fu-Tong Liu, Betty A. Wu-Hsieh

**Affiliations:** ^1^Graduate Institute of Immunology, National Taiwan University College of Medicine, Taipei, Taiwan; ^2^Institute of Biological Chemistry, Academia Sinica, Taipei, Taiwan; ^3^Department of Internal Medicine, National Taiwan University Hospital, Taipei, Taiwan; ^4^Institute of Biomedical Sciences, Academia Sinica, Taipei, Taiwan

**Keywords:** galectin-3, *Candida albicans*, neutrophil, reactive oxygen species, Syk kinase, TD139, M2 macrophage, monocyte/dendritic cell recruitment

## Abstract

Invasive candidiasis is a leading cause of nosocomial bloodstream infection. Neutrophils are the important effector cells in host resistance to candidiasis. To investigate the modulation of neutrophil fungicidal function will advance our knowledge on the control of candidiasis. While recombinant galectin-3 enhances neutrophil phagocytosis of *Candida*, we found that intracellular galectin-3 downregulates neutrophil fungicidal functions. Co-immunoprecipitation and immunofluorescence staining reveal that cytosolic gal3 physically interacts with Syk in neutrophils after *Candida* stimulation. *Gal3^−/−^* neutrophils have higher level of Syk activation as well as greater abilities to generate reactive oxygen species (ROS) and kill *Candida* than *gal3^+/+^* cells. While galectin-3 deficiency modulates neutrophil and macrophage activation and the recruitment of monocytes and dendritic cells, the deficiency does not affect the numbers of infiltrating neutrophils or macrophages. Galectin-3 deficiency ameliorates systemic candidiasis by reducing fungal burden, renal pathology, and mortality. Adoptive transfer experiments demonstrate that cell intrinsic galectin-3 negatively regulates neutrophil effector functions against candidiasis. Reducing galectin-3 expression or activity by siRNA or gal3 inhibitor TD139 enhances human neutrophil ROS production. Mice treated with TD139 have enhanced ability to clear the fungus. Our work unravels the mechanism by which galectin-3 regulates Syk-dependent neutrophil fungicidal functions and raises the possibility that blocking gal3 in neutrophils may be a promising therapeutic strategy for treating systemic candidiasis.

## Introduction

Galectin-3 (gal3) belongs to a family of evolutionarily highly conserved animal lectins. It is structurally unique amongst all the known galectins by consisting of a proline- and glycine-rich non-lectin domain in the N-terminal part, in addition to the C-terminal carbohydrate-recognition domains (CRD) (1). Gal3 binds to the *N*-acetyllactosamine motif of glycoproteins through its CRD and undergoes polymerization through its N-terminal part (1, 2). It is expressed in macrophages, dendritic cells, activated lymphocytes, epithelial cells, and neutrophils and found in both intracellular and extracellular compartments as well as on the cell surface (3). Recombinant gal3 has been reported to function extracellularly as a chemoattractant, a pro-apoptosis mediator, an opsonizing molecule, a phagocytosis enhancer, and a mediator that bridges hyperglycosylated mucin MUC2 and dendritic cells (2). Cell intrinsic gal3 has also been reported to function intracellularly as an anti-apoptosis mediator and a regulator for T cell activation, keratinocyte migration, and phagocyte cellular responses (4–8). Intracellular gal3 modulates cellular responses and tumor cell apoptosis by direct association with internalized dectin-1 (9), signaling molecule (10), adaptor protein (11), or apoptosis regulator (12). Point mutation at the CRD abolishes gal3 binding to Bax, thereby interfering its anti-apoptotic effect (12), showing that the CRD of gal3 is involved in interaction with proteins, but the interaction is carbohydrate independent.

Neutrophils express gal3 intracellularly although at lower levels compared to other innate cells (3, 13). *In vitro* studies showed that gal3 added exogenously improves the ability of neutrophils to migrate, phagocytose, and produce IL-8 and reactive oxygen species (ROS) (13–15). Studies of the role of intracellular gal3 in neutrophil functions are limited. By comparing *gal3^−/−^* to *gal3^+/+^* cells, gal3 is found to inhibit the neutrophil ROS response to phorbol myristate acetate or zymosan stimulation and regulate the life span of neutrophils upon *Toxoplasma gondii* infection (4). Thus, it appears that exogenously added gal3 and intrinsic gal3 have distinct effects on neutrophils.

*Candida albicans* is a commensal organism colonizing human mucosal surface and the skin. The commensal organism grows as yeast and germinates to invade the host (16). Neutropenic patients or individuals with neutrophil dysfunction are at higher risk for invasive candidiasis (17–19). Invasive candidiasis affects more than 250,000 people each year and leads to more than 50,000 deaths worldwide. Mortality among patients with invasive candidiasis can be as high as 40% even after receiving antifungal therapy (20). Study on candidemia cases revealed that time to initiate antifungal therapy has no impact on 30-day mortality (21), demonstrating that most patients do not respond to antifungal treatment. To develop better diagnostic biomarkers for patients with candidemia and better therapeutic strategy for disseminated candidiasis are important issues. In a mouse model of systemic candidiasis, *Candida* has marked tropism for kidney and brain (22). Mice with X-linked chronic granulomatous disease or with myeloperoxidase deficiency whose neutrophils are defective in producing ROS succumb to sublethal *Candida* infection (23). Depleting neutrophils exacerbates systemic candidiasis (24). These reports support the notion that neutrophils through producing ROS are responsible for clearing *Candida* infection in both humans and mice. The animal model of systemic candidiasis is a powerful tool to investigate the function of neutrophils against *Candida* infection. Whether and how cell intrinsic gal3 regulates host defense against candidemia has never been addressed.

While most studies on the role of gal3 in *Candida* emphasize its extracellular functions, we uncover its intracellular role in modulating signaling pathways. Extracellular gal3 mostly sends outside-in signals to regulate cell activation, we show a direct regulation of Syk kinase by cytosolic gal3 in neutrophil response to *Candida*. Gal3 physically interacts with Syk and negatively regulates its activation. By applying cell-permeable gal3 inhibitor TD139 on human neutrophils or administering it to mice with systemic candidiasis, our work raises the possibility that blockade of gal3 in neutrophils may be a promising therapeutic strategy for systemic *Candida* infection.

## Materials and Methods

### Fungus and Infection

*Candida albicans* strain SC5314 (ATCC MYA-2876) and clinical isolates from patients with bloodstream infection (CL 10-1, CA 09-1, CL 15-1, CA 01-1, CL 03-1, and CA 03-1, National Taiwan University Hospital) were used in this study. SC5314, clinical isolates, and GFP-expressing strain OG1 (25) were cultured at 30°C on yeast–peptone–dextrose (YPD) agar (DIFCO) plate. Mice were injected intravenously with *Candida* yeasts prepared in RPMI 1640 medium. *Candida* yeasts were opsonized in phenol red free HBSS containing 10% fresh mouse or human serum at room temperature for 30 min.

### Mouse Neutrophil Enrichment and Adoptive Transfer

Bone marrow cells were harvested from the femurs and suspended in dPBS buffer before overlaid on discontinuous percoll gradients (55, 62, and 81% in the order from top to bottom) (GE Healthcare). After centrifugation at 1,400 × *g* for 30 min, cells at the interface between 62 and 81% gradients were harvested and washed. Flow cytometric analysis showed that 92% of cells were CD11b^+^Ly6G^+^. For adoptive transfer, 5 × 10^6^ neutrophils isolated from *gal3^+/+^, gal3^−/−^*, and littermate mice were injected into recipient mice through the tail vein. Recipient mice were given *Candida* intravenously 1 h later.

### Human Neutrophil Isolation

Fresh peripheral venous bloods were collected from healthy volunteers with no history of candidiasis in BD Vacutainer^®^ Plus Plastic K2EDTA Tubes or serum separator tubes for different purposes. Neutrophils were isolated as described previously (26). Briefly, bloods were diluted in Mg^2+^- and Ca^2+^-free DPBS and overlaid on Ficoll-Paque solution (Ficoll-Paque™ PLUS, GE Healthcare). After centrifugation at 600 × *g* for 15 min, the pellet containing neutrophils and red blood cells (RBCs) was subject to dextran sedimentation (3%) to separate RBCs from neutrophils. Residual RBCs were lysed by lysis buffer. Cell viability was determined by trypan blue exclusion.

### Staining Surface and Cytosolic Gal3

Neutrophils from human or mice were stimulated with opsonized *Candida* for 5 and 15 min. Cells were stained with rat anti-gal3 and fluorochrome-conjugated goat anti-rat antibodies. After fixation in 4% PFA and permeabilization in saponin (Sigma)-containing perm/wash buffer, cells were stained with Alexa 647-rat anti-gal3 antibody. Samples were acquired by BD FACSCanto II and analyzed by BD FACSDiva software.

### Renal Infiltrating Cell Isolation and Immunostaining

Kidneys were cut into small pieces and treated with 0.2 mg/ml Liberase TM (Roche) at 37°C for 30 min. Pellets were resuspended in 45% percoll and overlaid on 81% percoll. Cells at the interface (45/81%) were collected after centrifugation. Cells stained positive for CD45 were taken as infiltrating cells. After exclusion of Ly6G^+^ neutrophils, cells were divided into three distinct populations according to the expression of MHC II and F4/80 (MHC II^+^F4/80^+^ as macrophages, MHC II^−^F4/80^+^ as monocytes, and MHC II^+^F4/80^−^ as dendritic cells). Detailed description is given in Methods in Supplementary Material.

### ROS Production

Bone marrow neutrophils were incubated in phenol red free HBSS containing 10 µM CM-H_2_DCFDA (Life Technologies) for 30 min. After replenishment with fresh HBSS or with HBSS containing kinase inhibitors, cells were stimulated with opsonized *Candida* yeasts (MOI = 0.5). Oxidative DCF was analyzed by flow cytometry. To assess ROS production by renal infiltrating neutrophils isolated from infected mice, neutrophils collected from percoll gradients were stained with CM-H_2_DCFDA and subject to flow cytometric analysis.

Human neutrophils were pretreated with mock (0.5% DMSO) or 250 μM of TD139 (3,3′-deoxy-3,3-bis(4-[*m*-fluorophenyl]-1H-1,2,3-triazol-1-yl)-thio-digalactoside) (27) for 4 h before addition of CM-H_2_DCFDA. Cells were then stimulated by opsonized *Candida* for 20 min before subject to flow cytometric analysis.

### Fungicidal Activity Assay

The assay was adopted from Methods in Molecular Biology (28). Briefly, *Candida* yeasts were grown in Sabouraud-dextrose broth (pH = 5.6) in 37°C overnight. Bone marrow neutrophils and CD45^+^Ly6G^+^ renal infiltrating cells were subject to killing assay in 96-well plate with 5 × 10^5^ cells and 1 × 10^4^ opsonized *Candida* yeasts. Wells containing opsonized *Candida* yeasts without neutrophils was used as control. Twenty minutes was allowed for phagocytosis after which supernatants in control wells and experimental groups were plated on YPD plate. The number of ingested yeasts (*N*_0h_) = the number of yeasts in control well − the number of uningested yeasts. The rest of the wells were incubated for another 1.5 h and the cells were lysed to release intracellular yeasts. The lysates were plated. % Killing = (*N*_0h_ − *N*_1.5h_)/*N*_0h_ × 100%, where *N* = the number of intracellular *Candida*.

### Co-Immunoprecipitation

Five million neutrophils were stimulated with opsonized *Candida*. Cells were lysed in 500 µl of non-denaturing lysis buffer. Cell lysates were mixed with 1.5 µg of mouse anti-gal3 antibody (B2C10) or 1 µg of anti-Syk polyclonal antibody at 4°C overnight. The mixture was mixed with 50 µl of agarose beads slurry under rotary agitation at 4°C for 4 h. Lysate beads mixture was washed with IP washing buffer. Beads were then mixed with 25 µl of sample buffer and boiled to elute immunoprecipitates.

### Quantification of Fungal Load

Kidney and brain were homogenized in a tissue grinder with 1 ml of RPMI 1640 medium. One to ten serial dilutions were made and 0.1 ml was plated on YPD agar. Colonies were counted after incubation at 30°C for 2 days.

### *Lgals3* Gene Knockdown in Human Neutrophils

Three million human neutrophils were premixed with Nucleofector^®^ Solution (Lonza) plus supplement in total volume of 100 µl followed by addition of 2 µM of scrambled siRNA or siRNA targeting gal3 (Santa Cruz Biotechnology). Cells were transfected by using Nucleofector^®^ Program Y-001 in the nucleofector II device and incubated in RPMI complete medium for 7 h before further experiment. Knockdown efficiency was about 47%. Cell viability was determined by trypan blue exclusion.

### Statistics

Mann–Whitney test (non-parametric analysis) was used to compare the difference between two groups. Mortality after *Candida* infection was analyzed by log-rank and Fisher’s exact test. Statistical significance was defined as *p* < 0.05.

### Ethics Statement

Human study was approved by the Institutional Review Board of the National Taiwan University Hospital (Permit Number: 201103121IRB, 201401074INA). All written informed consents were received from participants prior to inclusion in this study. Mouse study was carried out in strict accordance with the recommendations in the Guidebook for the Care and Use of Laboratory Animals, The Third Edition, 2007, published by The Chinese-Taipei Society of Laboratory Animal Sciences. The experimental protocol was approved by the Committee on the Ethics of Animal Experiments of the National Taiwan University College of Medicine (Permit Number: 20120031, 20130238, and 20140304).

## Results

### Dynamics of gal3 Expression after Neutrophils Encountering Opsonized *Candida*

**Figure 1:**
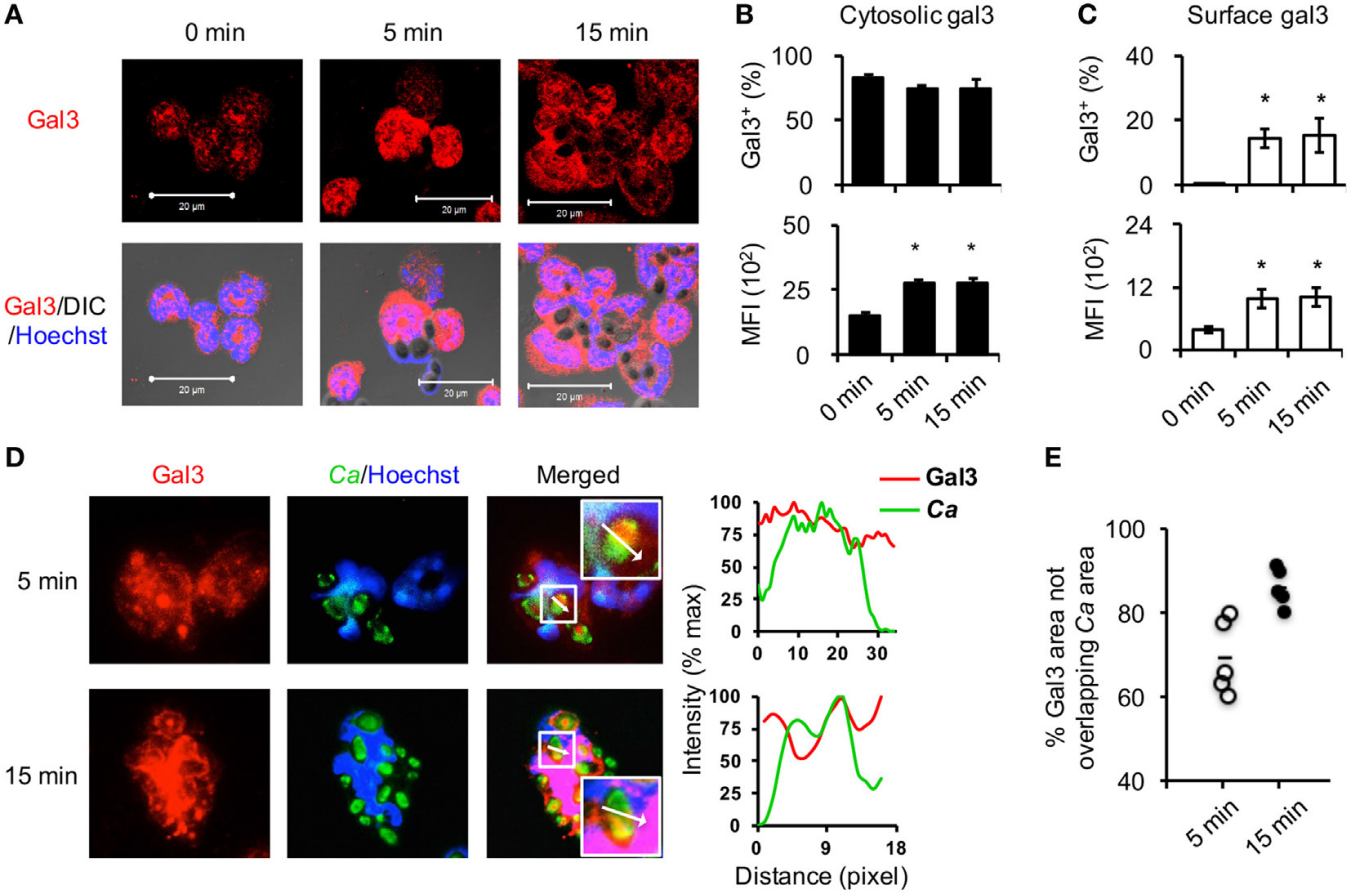
**Opsonized *Candida* upregulates cytosolic gal3 expression in neutrophils**. Bone marrow neutrophils were stimulated with opsonized *Candida* SC5314 **(A–C)** or GFP^+^ OG1 **(D,E)** for 5 or 15 min. Zero minutes represent unstimulated cell controls incubated in serum-containing medium for 15 min. **(A)** Cells were cytospun, permeabilized, and stained for gal3 (red) and nucleus (blue) before confocal microscopic analysis for gal3 expression. DIC, differential interference contrast. **(B,C)** Neutrophils were stained with rat anti-gal3 and PE-goat anti-rat antibodies, fixed in 4% PFA, permeabilized, and then stained with Alexa 647-rat anti-gal3 antibody. The % of cells expressing cytosolic gal3^+^ **(B)** and surface gal3^+^ **(C)** in total cell population are shown in *upper panels*. The levels of cytosolic **(B)** and surface gal3 **(C)** expression are shown as mean fluorescence intensity (MFI) in *lower panels*. Data are presented as mean ± SD. *p < 0.05, as analyzed by Mann–Whitney test. *n* = 3. **(D,E)** Neutrophils were allowed to ingest GFP^+^ OG1 *Candida* (green) and stained for gal3 (red) and nucleus (blue) before subjecting to fluorescence microscopic analysis. **(D)** The intensity of fluorochromes along the white arrow in the merged image is shown in the histogram on the right. **(E)** The % of gal3^+^ area (red) that is not overlapped with *Candida* (green) in the whole gal3^+^ area (red) (180,760 µm^2^ in average) at 5 and 15 min after encounter was analyzed by Metamorph software. Each dot represents one photo image. *n* = 5.

Previous studies revealed that recombinant gal3 positively regulates neutrophil functions when added exogenously, here we focused our study on the role of cell intrinsic gal3 in neutrophils. Fluorescence microscopic (Figure 1A) and flow cytometric analyses (Figures 1B,C) showed that above 80% of unstimulated neutrophils expressed low levels of gal3 in the cytosol and stimulation with opsonized *Candida* increased intracellular gal3 expression as early as 5 min after stimulation (Figure 1B). While dendritic cells and monocytes/macrophages constitutively express gal3 on the surface (29, 30), gal3 was not detected on the surface of unstimulated neutrophils (Figure 1C). Stimulation by *Candida* induced gal3 redistribution to the cell surface in about 20% of neutrophils (Figure 1C). It was noted that intracellular gal3 was diffusely distributed (Figure 1D) and 70.0 ± 8.8% and 85.9 ± 4.6% of the intracellular gal3 at 5 and 15 min time points, respectively, did not colocalize with opsonized *Candida* (Figures 1D,E). By tracking the movement of cytosolic gal3 in neutrophils upon encountering GFP-expressing *Candida*, we found that gal3 moved toward engulfed *Candida* (Figure S1 in Supplementary Material). At the point when gal3 was closest to *Candida* (7.56 min), it still did not associate with the organism (Figure S1 and Video 1 in Supplementary Material). These results together indicate that stimulation by opsonized *Candida* upregulates gal3 expression in neutrophils and that intracellular gal3 does not bind to the engulfed organism.

### Gal3 Negatively Regulates Complement Receptor 3 (CR3) Downstream ROS-Dependent Killing of *Candida* in Neutrophils

**Figure 2:**
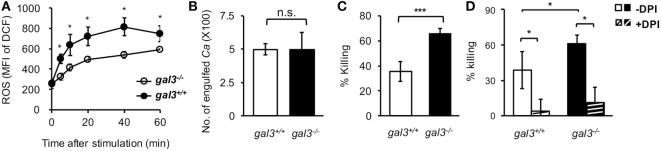
**Gal3 negatively regulates complement receptor 3 downstream reactive oxygen species (ROS)-dependent killing of *Candida* by neutrophils**. *Gal3^+/+^* (WT), *gal3^−/−^, Itgam^−/−^*, and *gal3^−/−^Itgam^−/−^* neutrophils were allowed to ingest opsonized *Candida* yeasts. **(A)** The level of ROS production shown as mean fluorescence intensity (MFI) of oxidized DCF fluorescence. Zero minutes represent the MFI of unstimulated control incubated in serum-containing medium. *n* = 7 pooled from two independent experiments in panel **(A)**. **(B)** Twenty minutes was allowed for phagocytosis. The number of *Candida* (*Ca*) engulfed by neutrophils is shown as CFU. **(C,D)** The ability of neutrophils to kill opsonized *Candida* is shown as % killing. Neutrophils were treated with or without NADPH inhibitor diphenyleneiodonium (DPI, 10 µM) for 30 min before addition of opsonized yeasts **(D)**. DPI was left in the culture over the entire period. *n* = 7–8 pooled from three independent experiments in panels **(B,C)**; and *n* = 3 in panel **(D)**. Data are presented as mean ± SD. *p < 0.05; ***p < 0.005; n.s., not significant, as analyzed by Mann–Whitney test. See also Figure S1 in Supplementary Material.

**Figure 3:**
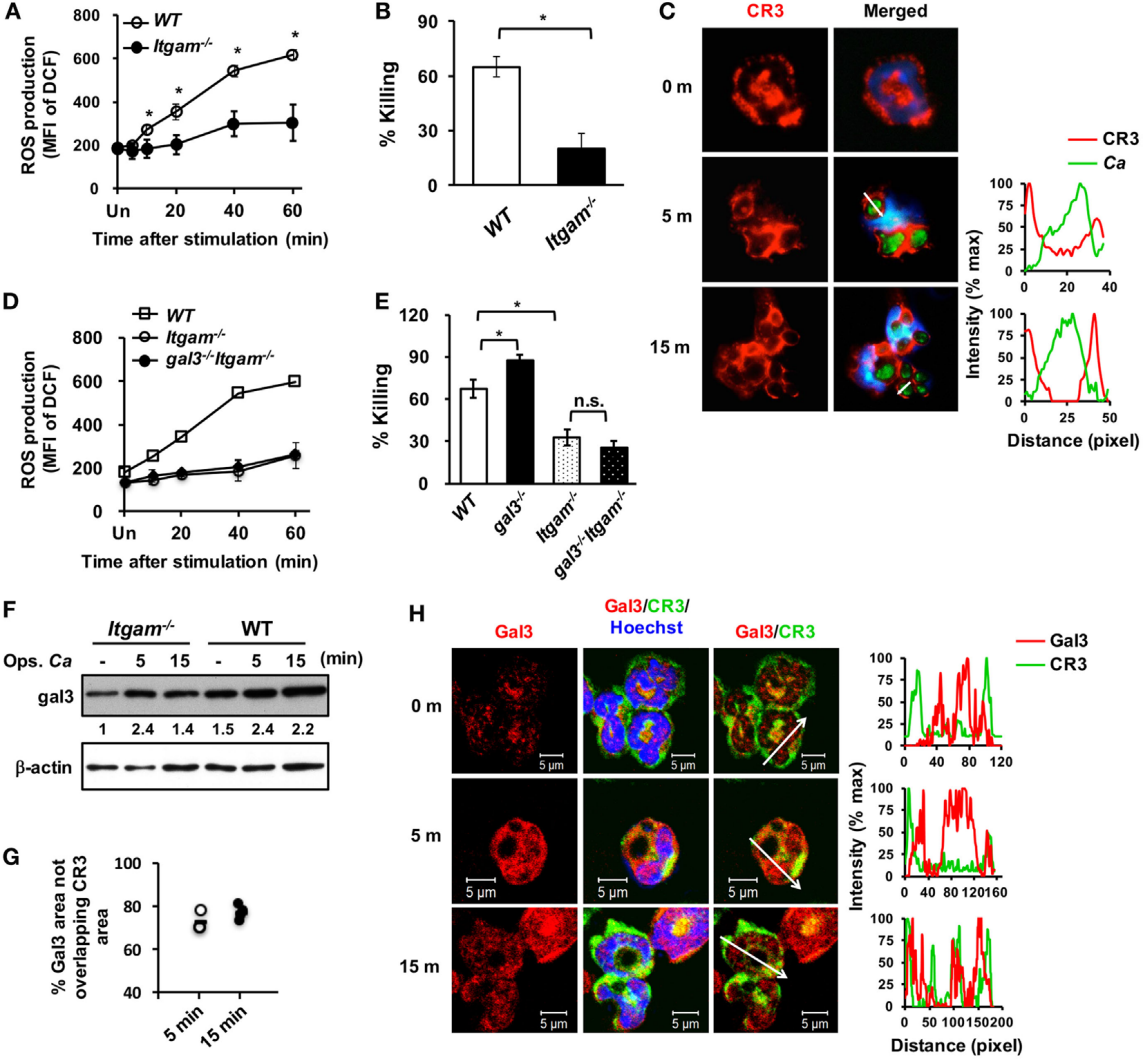
**Gal3 negatively regulates complement receptor 3 (CR3)-mediated killing of *Candida* by neutrophils**. Neutrophils isolated from WT, *gal3^−/−^, Itgam^−/−^, gal3^−/−^Itgam^−/−^* mice were allowed to ingest opsonized *Candida* yeasts. **(A,D)** The level of reactive oxygen species (ROS) production is shown as mean fluorescence intensity (MFI) of oxidized DCF fluorescence. *n* = 3 in panel **(A)**; *n* = 4 in panel **(D)**. Experiment was performed twice. **(B,E)** The ability of neutrophils to kill opsonized *Candida* is shown as % killing. *n* = 3 in panel **(B)**; and *n* = 4 in panel **(E)**. Experiment was performed twice. Data are presented as mean ± SD. *p < 0.05; n.s., not significant, as analyzed by Mann–Whitney test. **(C)** Confocal fluorescence microscopic analysis to localize CR3 (red) and *Candida* OG1 (green). **(F)** Gal3 expression in *Itgam^−/−^* and WT neutrophils stimulated with opsonized *Candida* (Ops. *Ca*) was detected by Western blot. β-actin was used as a loading control. The numbers below the gel denote the relative intensity of gal3. Experiment was performed three times. **(G,H)** Confocal microscopy analysis to localize gal3 (red) and CR3 (green). The intensity of different fluorochromes along the white arrow in the merged **(C)** and Gal3/CR3 **(H)** images is shown in the histogram on the right. **(G)** Metamorph software was employed to determine the percentages of non-localization between gal3 and CR3 in the whole gal3 area in image in panel **(H)** (56,444 µm^2^ in average). Each dot represents one photo. *n* = 4.

To unravel the role of upregulated gal3 in neutrophils, *gal3^+/+^* and *gal3^−/−^* neutrophils were allowed to ingest opsonized *Candida*, and the subsequent cellular response was assessed. We found that *gal3^−/−^* neutrophils produced higher levels of ROS than *gal3^+/+^* cells (Figure 2A). While *gal3^−/−^* and *gal3^+/+^* cells were comparable in their abilities to engulf *Candida* (Figure 2B), *gal3^−/−^* neutrophils killed ingested *Candida* more efficiently than *gal3^+/+^* cells (Figure 2C). Moreover, suppressing ROS completely abolished killing of ingested *Candida* yeasts by both *gal3^+/+^* and *gal3^−/−^* neutrophils (Figure 2D). These results indicate that both *gal3^+/+^* and *gal3^−/−^* neutrophils kill ingested *Candida* through a ROS-dependent mechanism and that gal3 suppresses neutrophil fungicidal activity through inhibiting ROS production. Interestingly, CR3-deficient (*Itgam^−/−^*) neutrophils were significantly less efficient in producing ROS (Figure 3A) and killing of opsonized *Candida* (Figure 3B) than WT cells, but such functions were not affected in dectin-1-deficient (*clec7a^−/−^*) neutrophils (Figures S2A,B in Supplementary Material). Immunofluorescence staining revealed that CR3 congregated around but did not colocalize with engulfed *Candida* (Figure 3C). Thus, it appears that ROS production as a result of CR3 engagement is responsible for neutrophil fungicidal activity. In addition, gal3 deficiency promoted the abilities of dectin-1-deficient neutrophils to produce ROS (Figure S2C in Supplementary Material) and kill *Candida* (Figure S2D in Supplementary Material), whereas the presence or absence of gal3 did not make any difference in ROS production (Figure 3D) and the ability to kill *Candida* by *Itgam^−/−^* cells (Figure 3E). It reveals that the suppressive effect of gal3 on neutrophil anti-*Candida* function is through CR3. It is worth noting that gal3 expression increased by twofold in *Itgam^−/−^* neutrophils after stimulation by opsonized *Candida* compared to that without stimulation (Figure 3F), revealing that gal3 upregulation is independent of CR3. Moreover, we found that majority (>72%) of the intracellular gal3 in the cell population we tracked did not colocalize with CR3 (Figures 3G,H). These results together suggest that although neutrophil production of ROS is through CR3 engagement with *Candida*, gal3 does not directly interact with CR3.

### Gal3 Physically Interacts with Syk and Downregulates CR3 Downstream Syk-Mediated ROS Production

**Figure 4:**
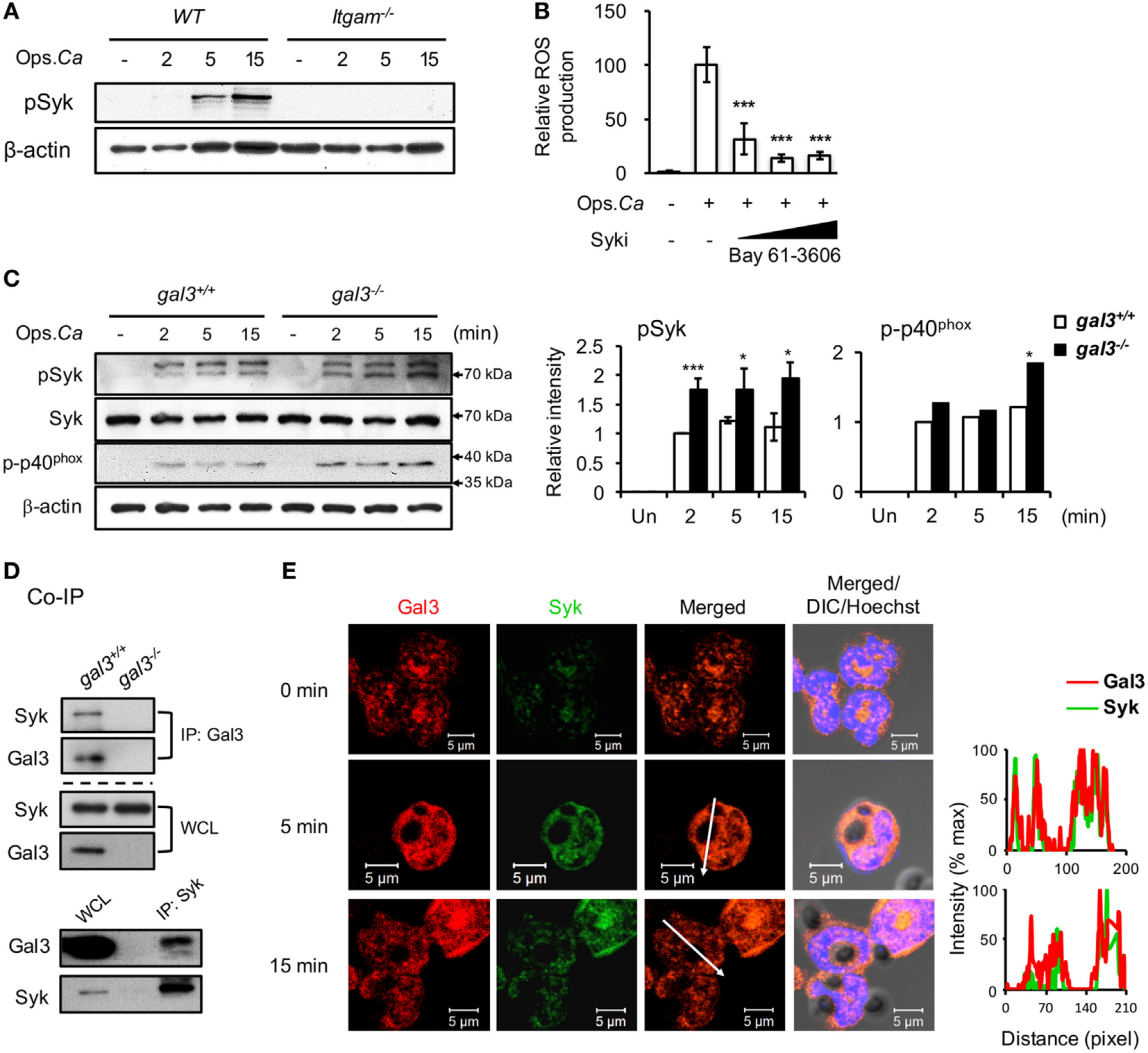
**Gal3 negatively regulates complement receptor 3 downstream Syk-mediated neutrophil reactive oxygen species (ROS) response to *Candida***. *Gal3^+/+^* (WT), *gal3^−/−^*, and *Itgam^−/−^* neutrophils were stimulated with opsonized *Candida* (Ops. *Ca*). **(A)** Cell lysates were collected at different time points after stimulation and subjected to Western blot analysis with anti-p-Syk antibody. β-actin was used as a loading control. **(B)** WT cells were pretreated with 2, 10, and 50 µM of Syk inhibitor before stimulation. The levels of ROS produced by neutrophils at 20 min after stimulation were analyzed by flow cytometry. Relative ROS production was calculated by dividing mean fluorescence intensity of oxidized DCF fluorescence in *Candida*-stimulated cells by that in untreated cells. *n* = 6 as pooled from two independent experiments. **(C)** Cell lysates were collected at different time points after stimulation and subjected to Western blot analysis with anti-p-Syk, -Syk, or -p-p40^phox^ antibodies. β-actin was used as a loading control. Relative intensity of p-Syk (*n* = 5) and p-p40^phox^ (*n* = 2) at different time points are shown in the *right panel*. Data are presented as mean ± SEM. *p < 0.05, ***p < 0.005, as analyzed by Mann–Whitney test. **(D)** Whole cell lysates (WCL) were immunoprecipitated with anti-gal3 (IP: Gal3, *upper panel*) and anti-Syk (IP: Syk, *lower panel*) antibodies. Precipitates were subjected to Western blot analysis with anti-Syk and anti-gal3 antibodies. Experiments were performed three times. Data from one representative experiment are shown. **(E)** Confocal microscopy image of gal3 (red) and Syk (green) in neutrophils after stimulation by *Candida*. The intensity of different fluorochromes along the white arrow in the merged image is shown in the histogram on the right. See also Figure S1 in Supplementary Material.

It is reported that CR3 engagement activates neutrophil killing of *Candida* through Syk signaling (31). Our data showed that the levels of pSyk in CR3-deficient neutrophils were completely abolished after stimulation (Figure 4A) while that in dectin-1-deficient cells were comparable to WT cells (Figure S2E in Supplementary Material). Treatment with Syk inhibitor Bay 61-3606 dose-dependently reduced neutrophil ROS production (Figure 4B). Importantly, the levels of pSyk and p-p40^phox^ were significantly higher in *gal3^−/−^* than in *gal3^+/+^* neutrophils after stimulation (Figure 4C). Reciprocal co-immunoprecipitation (Figure 4D) and immunofluorescence staining (Figure 4E) revealed that gal3 directly interacted with Syk after stimulation. Therefore, ROS production by neutrophils in response to opsonized *Candida* is Syk-dependent and gal3, by modulating Syk activation, regulates CR3 downstream signaling pathway(s) leading to ROS production.

### Gal3 Deficiency Enhances the Fungicidal Activity of Renal Infiltrating Neutrophils

**Figure 5:**
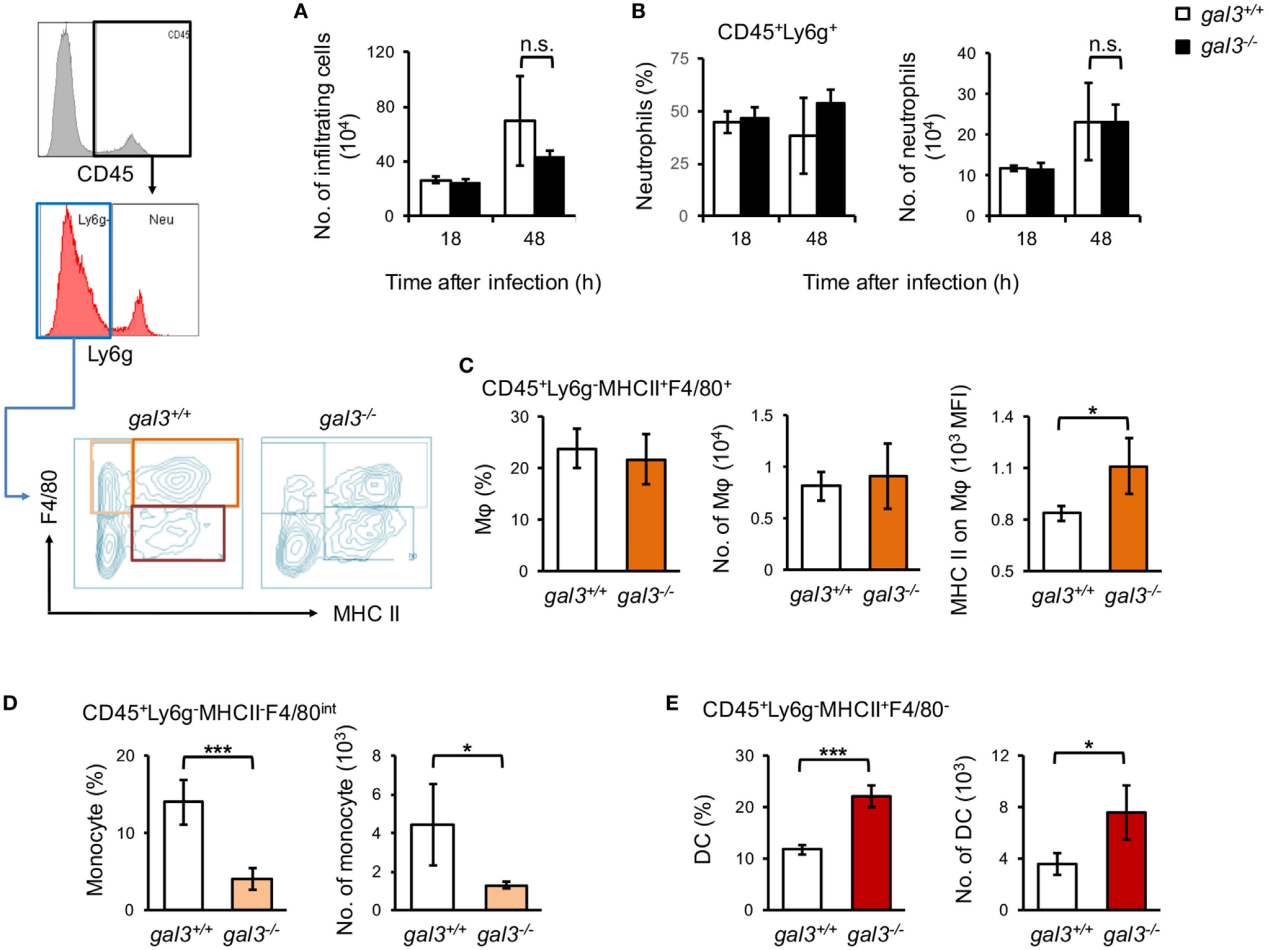
**Gal3 regulates renal macrophage activation, monocytes, and dendritic cell recruitment in systemic *Candida* infection**. *Gal3^+/+^* and *gal3^−/−^* mice were infected intravenously with 5 × 10^5^ of *Candida*. Kidneys were collected from infected mice at 18 and 48 h after infection. Renal infiltrating cells were isolated and stained for lineage specific markers as indicated. Total number of renal infiltrating cells (CD45^+^ cells) **(A)**, the percentages and numbers of neutrophils (CD45^+^Ly6G^+^) **(B)**, macrophages (Mφ, CD45^+^Ly6G^−^MHCII^+^F4/80^+^) **(C)**, monocytes (CD45^+^Ly6G^−^MHCII^−^F4/80^+^) **(D)**, and dendritic cells (CD45^+^Ly6G^−^MHCII^+^F4/80^−^) **(E)** in the kidney are shown. The levels of MHC class II expression on macrophages are shown as mean fluorescence intensity **(C)**. *n* = 3. Data are presented as mean ± SD. *p < 0.05, ***p < 0.005, n.s., not significant, as analyzed by Mann–Whitney test.

Previous studies revealed the role of gal3 in cell recruitment *in vivo* (32, 33). To address whether gal3 also regulates cell infiltration in systemic candidiasis, we analyzed renal infiltrating neutrophils (CD45^+^Ly6G^+^), macrophages (CD45^+^Ly6G^−^MHCII^+^F4/80^+^), monocytes (CD45^+^Ly6G^−^MHCII^−^F4/80^+^), and dendritic cell (CD45^+^Ly6G^−^MHCII^+^F4/80^−^) populations (34). The results showed that gal3 deficiency did not affect the recruitment of neither neutrophils nor macrophages (Figures 5A–C). Gal3 deficiency, however, reduced monocyte (Figure 5D; Figure S4A in Supplementary Material) but enhanced dendritic cell (Figure 5E; Figure S4A in Supplementary Material) infiltration. In addition, the levels of chemokines were not different between *gal3^−/−^* and *gal3^+/+^* mice after infection (Figure S3 in Supplementary Material). Interestingly, we found that renal infiltrating macrophages in *gal3^−/−^* mice expressed higher levels of MHC II (Figure 5C), iNOS, and lower CD206 than in *gal3^+/+^* mice (Figure S4B in Supplementary Material).

**Figure 6:**
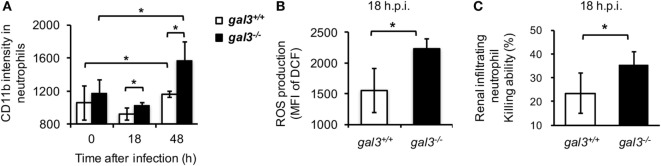
**Gal3 deficiency enhances infiltrating neutrophil anti-*Candida* functions**. *Gal3^+/+^* and *gal3^−/−^* mice were infected intravenously with 5 × 10^5^ of *Candida*. **(A)** Blood was collected from naïve mice (0) or mice at 18 h and 48 h after infection. The levels of CD11b expression on CD45^+^CD11b^+^Ly6G^+^ peripheral blood neutrophils are presented as mean fluorescence intensity (MFI). *n* = 3. **(B,C)** CD45^+^CD11b^+^Ly6G^+^ cells were sorted from total infiltrating cells in the kidney at 18 h after infection. The levels of reactive oxygen species (ROS) production **(B)** and their ability to kill **(C)** were determined. *n* = 3. Data are presented as mean ± SD. *p < 0.05, as analyzed by Mann–Whitney test.

Peripheral blood neutrophils from infected *gal3^−/−^* were compared to those from *gal3^+/+^* mice for the levels of activation marker CD11b (35). Results in Figure 6A showed that CD11b on neutrophils from *gal3^−/−^* mice were significantly higher than cells from *gal3^+/+^* mice after infection (Figure 6A). Moreover, renal infiltrating neutrophils of infected *gal3^−/−^* mice produced significantly higher amount of ROS (Figure 6B) and their ability to kill *Candida* (Figure 6C) was higher than cells of *gal3^+/+^* mice. These results demonstrated that gal3 negatively regulates neutrophil functions not only *in vitro* but also *in vivo*.

### *Gal3^−/−^* Mice Are More Resistant to Systemic *Candida* Infection

**Figure 7:**
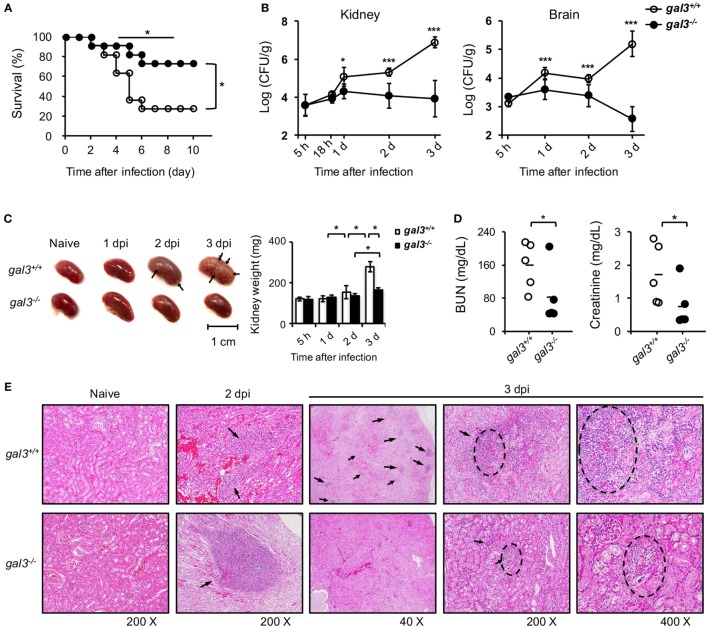
**Gal3 deficiency ameliorates *Candida* infection**. *Gal3^+/+^* and *gal3^−/−^* mice were infected intravenously with 5 × 10^5^ of *Candida*. Kidneys were collected from uninfected (naïve) and infected mice at different time points after infection (dpi). **(A)** The survival of infected mice was followed until day 10 after infection. *n* = 11. Data are pooled from three independent experiments. *p < 0.05, as analyzed by log-rank and Fisher’s exact test. **(B)** Fungal burden in the kidney is shown as Log_10_ CFU per gram of tissue. *n* = 6. Data are pooled from two independent experiments and presented as mean ± SD. **(C)** Kidneys were collected from uninfected and infected mice on different days after infection (dpi). *Left*, arrows point to abscess lesions; *right*, kidney weight. *n* = 6. Data are pooled from two independent experiments and presented as mean ± SD. **(D)** The levels of blood urea nitrogen and creatinine in mouse sera on day 2 after infection. *n* = 5. Data are pooled from two independent experiments. *p < 0.05; ***p < 0.005, as analyzed by Mann–Whitney test. **(E)** Kidneys were collected and fixed in formalin before H&E stain. The magnification is 200× for sections from naïve mice and mice at day 2 after infection and 40×, 200×, and 400× for sections from mice at day 3 after infection. Arrows point to inflammatory foci. *n* = 3. See also Figures S2 and S3 in Supplementary Material.

Consistent with higher neutrophil fungicidal functions, *gal3^−/−^* mice had better survival (Figure 7A) and lower fungal burdens in kidneys and brain (Figure 7B) than *gal3^+/+^* mice when systemically infected with *Candida*. While *gal3^+/+^* mice sustained multiple abscesses in the kidney with their kidney weights being about 2.3-fold of that of uninfected mice, *gal3^−/−^* mice exhibited only minimal gross renal pathology and relatively less change in kidney weights (Figure 7C). The creatinine and blood urea nitrogen levels were also significantly lower in *gal3^−/−^* mice than in *gal3^+/+^* mice (Figure 7D). Both *gal3^+/+^* and *gal3^−/−^* mice exhibited chronic-active nephritis and pyelonephritis with inflammatory infiltrates on day 2 after infection. Starting on day 3, nephritis became severe in *gal3^+/+^* mice, but remained minimal/slight in *gal3^−/−^* mice (Figure 7E), indicating that severe inflammation and tissue damage occurred to *gal3^+/+^* but not to *gal3^−/−^* mice. Additionally, while hyphal/pseudohyphal forms of *Candida* were found in both infected *gal3^+/+^* and *gal3^−/−^* mice on 18 h after infection, these forms increased and persisted in *gal3^+/+^* mice, but became almost completely absent in *gal3^−/−^* mice by 3 days after infection (Figure S5 in Supplementary Material). These results together show that gal3 deficiency ameliorates systemic candidiasis by reducing fungal burdens, renal pathology, and mortality.

### Neutrophil Intrinsic gal3 Suppresses Host Defense against *Candida*

**Figure 8:**
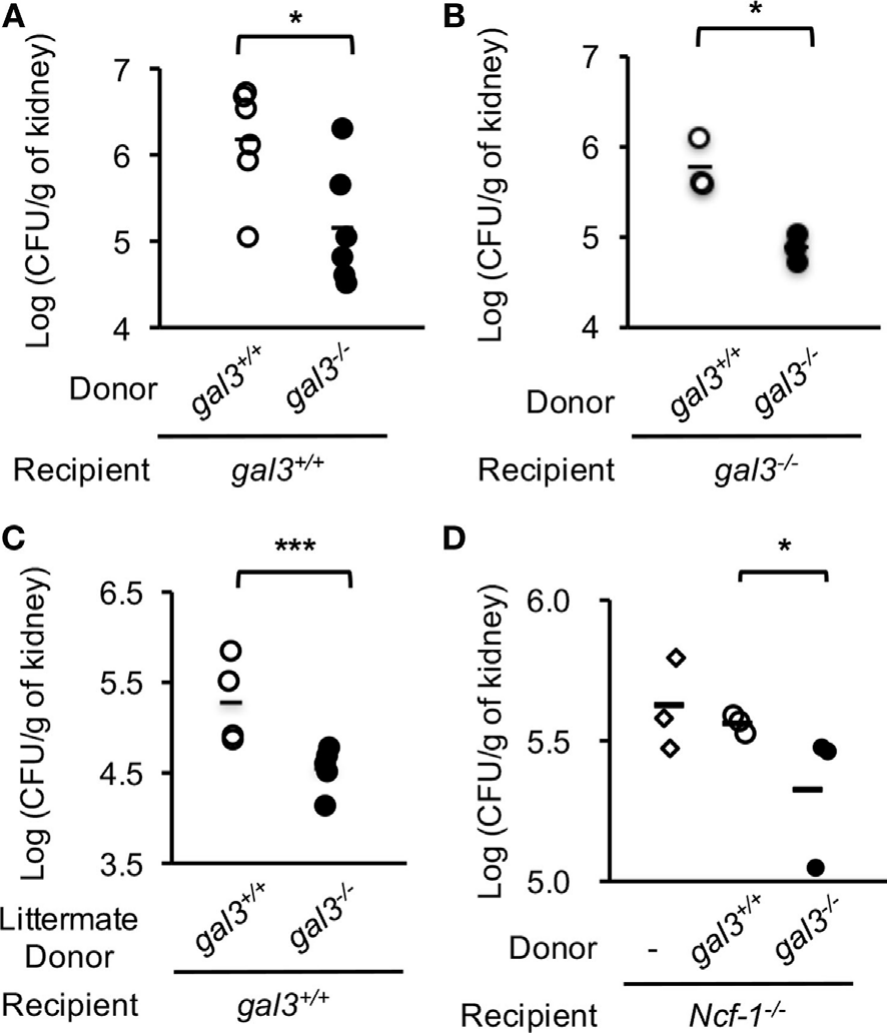
**Adoptive transfer of *gal3^−/−^* neutrophils reduces fungal burden in recipient mice**. Bone marrow neutrophils were collected from *gal3^+/+^* and *gal3^−/−^* mice **(A,B,D)** or *gal3^−/−^* mice and their littermates **(C)**. Five million cells were adoptively transferred into *gal3^+/+^* **(A,C)**, *gal3^−/−^* **(B)**, and *ncf-1^−/−^* **(D)** recipients separately. Recipient mice were infected with 5 × 10^5^ **(A–C)** or 1 × 10^3^ **(D)** of *Candida* yeast cells 1 h later. Fungal burdens in kidneys from recipient mice on 2 days after infection are shown as Log_10_ (CFU per gram of kidney). *n* = 6 pooled from two independent experiments in panel **(A)**; *n* = 3 in panel **(B)**; *n* = 5 in panel **(C)**; and *n* = 3 in panel **(D)**. *p < 0.05; ***p < 0.005, as analyzed by Mann–Whitney test.

Adoptive transfer experiments were performed to investigate whether gal3 regulates neutrophil functions *in vivo* is a cell intrinsic event. We found that adoptively transferred *gal3^−/−^* neutrophils cleared *Candida* infection more efficiently than *gal3^+/+^* neutrophils whether the recipient mice were *gal3^+/+^* (Figure 8A) or *gal3^−/−^* (Figure 8B). Transfer of neutrophils from littermate donors also demonstrated the suppressive effect of gal3 on neutrophils in clearing *Candida* (Figure 8C). When neutrophil cytosolic factor 1-deficient (*ncf-1^−/−^*) mice were used as recipients, transfer of *gal3^−/−^* neutrophils also resulted in significantly less fungal burdens than transfer of *gal3^+/+^* cells (Figure 8D). The results confirm that it is ROS produced by transferred neutrophils but not that by cells in the recipient mice that mediates host defense against *Candida*. Taken together, these results indicate that cell intrinsic gal3 regulates neutrophil anti-*Candida* function.

### Gal3 Negatively Regulates Primary Human Neutrophil ROS Response to Opsonized *Candida*

**Figure 9:**
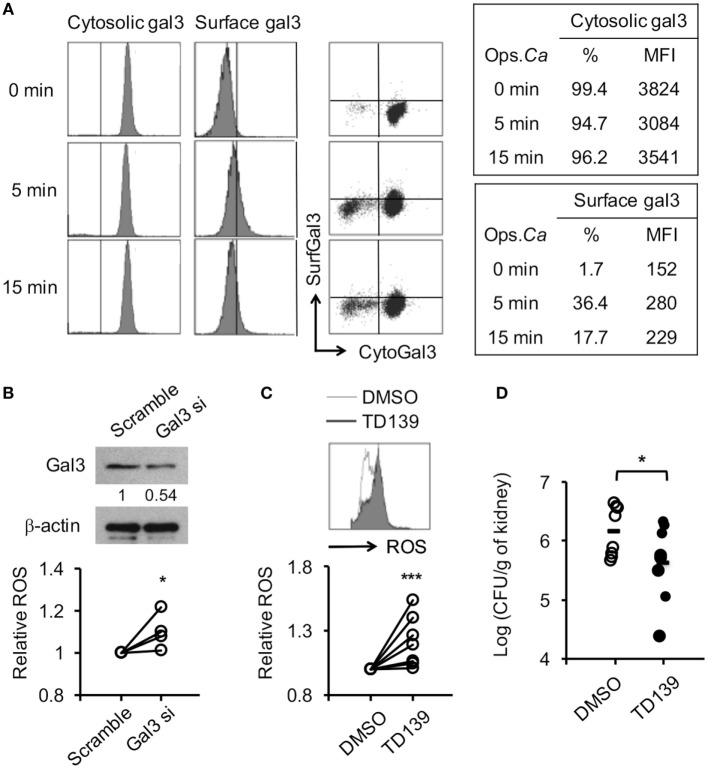
**Reducing gal3 expression or activity enhances human neutrophil reactive oxygen species (ROS) response to opsonized *Candida* and host resistance to *Candida* infection**. **(A–C)** Human neutrophils isolated from peripheral blood of healthy volunteers were allowed to ingest opsonized (in 5% human serum) *Candida* yeasts. **(A)** The % of cells expressing cytosolic gal3^+^ or surface gal3^+^ in total cells. **(B,C)** Cells were transfected with gal3 (Gal3 si) or scrambled (Scramble) siRNA **(B)** or pretreated with mock (0.5% DMSO) or 250 µM of TD139 for 4 h **(C)** before stimulation with opsonized *Candida*. The level of ROS production by neutrophils was analyzed by flow cytometry at 20 min after stimulation. Relative ROS production was calculated by dividing the mean fluorescence intensity (MFI) of cells treated with Gal3 siRNA TD139 and by that treated with scrambled siRNA and in DMSO, respectively. The levels of gal3 protein in cells transfected with gal3 or scrambled siRNA were detected by Western blot to determine the efficiency of gal3 siRNA knockdown. β-actin was used as a loading control. The numbers below the gel denote the relative intensity of gal3. **(D)** *Gal3^+/+^* mice were infected with 5 × 10^5^ of *Candida* yeast cells intravenously. After 30 min, infected mice were injected with 1.5% DMSO (DMSO) or 300 µg of TD139 in 200 µl of RPMI medium intraperitoneally. Fungal burdens in kidneys from infected mice on 2 days after infection are shown as Log_10_ (CFU per gram of kidney) *n* = 4 in panel **(B)**; *n* = 7 in panel **(C)**; and *n* = 8 in panel **(D)**. *p < 0.05; ***p < 0.005, as analyzed by Mann–Whitney test.

Human neutrophils were like mouse neutrophils (Figure 1A) constitutively expressed gal3 in the cytosol but not on the surface (Figure 9A). Surface gal3 became detectable as early as 5 min after encountering opsonized *Candida* (Figure 9A). Interestingly, silencing *Lgals3* in primary human neutrophils by siRNA significantly enhanced ROS production (Figure 9B). Treatment with gal3 inhibitor TD139, a derivative of thiodigalactoside, augmented human neutrophil ROS production (Figure 9C) and reduced fungal burden in mice with systemic candidiasis (Figure 9D). These results demonstrate that gal3 is a negative regulator of human neutrophil ROS response and point to the possibility of employing TD139 as a treatment strategy to restore neutrophil functions against *Candida* infection.

### The Negative Effect of gal3 on Neutrophil ROS Production Is Generalizable to Clinical *C. albicans* Isolates

**Figure 10:**
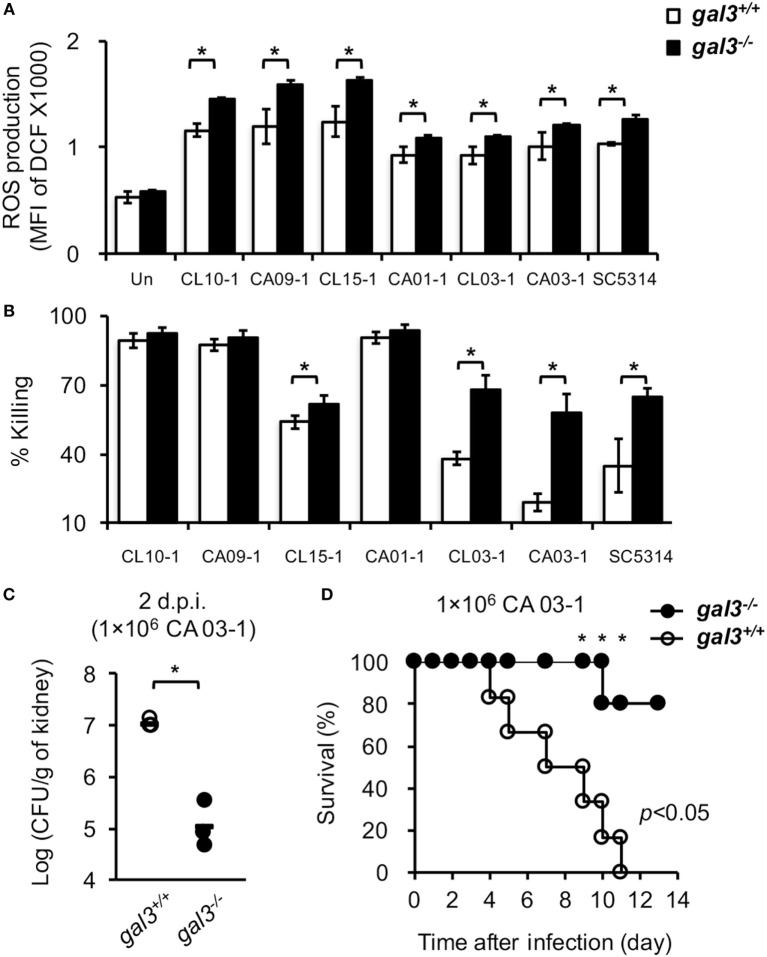
**The effect of gal3 on neutrophil response to *Candida albicans* clinical isolates**. **(A,B)** Bone marrow neutrophils isolated from *gal3^+/+^* and *gal3^−/−^* mice were allowed to ingest opsonized *C. albicans* clinical isolates CL 10-1, CA 09-1, CL 15-1, CA 01-1, CL 03-1, CA 03-1, and SC 5314. **(A)** The levels of reactive oxygen species (ROS) production are shown as mean fluorescence intensity (MFI) of oxidized DCF fluorescence. *n* = 4. **(B)** The abilities of neutrophils to kill opsonized *Candida* are shown as % killing. *n* = 4. Data are presented as mean ± SD. *p < 0.05, as analyzed by Mann–Whitney test. **(C,D)** *Gal3^+/+^* and *gal3^−/−^* mice were infected intravenously with 1 × 10^6^ of clinical isolate CA 03-1. **(C)** Fungal burdens in kidneys on day 2 after infection are presented as Log_10_ (CFU per gram of kidney). *Gal3^+/+^, n* = 4; and *gal3^−/−^, n* = 3. *p < 0.05, as analyzed by Mann–Whitney test. **(D)** The survival of infected mice was followed until day 10 after infection. *Gal3^+/+^, n* = 6; and *gal3^−/−^, n* = 5. *p < 0.05, as analyzed by log-rank and Fisher’s exact test.

We tested *C. albicans* isolates from six patients with bloodstream candidiasis and showed that all six clinical isolates induced WT neutrophil ROS production (Figure 10A). Although the levels of ROS induced by different isolates varied, gal3 deficiency enhanced its production (Figure 10A). Isolates CL 15-1, CL 03-1, and CA 03-1, like SC5314, were more susceptible to killing by *gal3^−/−^* neutrophils than by *gal3^+/+^* neutrophils (Figure 10B), while isolates CL 10-1, CA 09-1, and CA 01-1 were highly and equally susceptible (>90%) to killing by both *gal3^−/−^* and *gal3^+/+^* neutrophils (Figure 10B). Consistent with the observation about the role of gal3 in systemic SC5314 infection, *gal3^−/−^* mice were more efficient than *gal3^+/+^* mice in clearing CA 03-1 infection (Figure 10C) and had better survival (Figure 10D). Thus, gal3 negatively regulates neutrophil ROS production in response to not only SC5314 but also other clinical isolates and the negative effect of gal3 on neutrophil killing is more pronounced on otherwise “hard-to-kill” isolates.

## Discussion

In this study, we show that gal3 serves as a negative regulator of human and mouse neutrophil anti-*Candida* functions. Gal3 is expressed in the cytosol of both unstimulated human and mouse neutrophils. Responding to opsonized *Candida* stimulation, gal3 became detectable on the cell surface. While engulfed *Candida* is wrapped in phagosome (36), cytosolic gal3 does not directly contact the organism. Rather, it interacts with Syk and modulates its phosphorylation, through which it suppresses neutrophil anti-*Candida* function. Results of adoptive transfer experiments clearly demonstrates that cell intrinsic gal3 in neutrophils dampens host resistance to candidiasis. The negative effect of gal3 in primary human neutrophils is rescued by TD139 treatment and siRNA knockdown and demonstrable in neutrophil ROS response to clinical isolates of *C. albicans*. This study uncovers the immunomodulatory role of cell intrinsic gal3 in both human and mouse neutrophils and in host defense against systemic candidiasis in a mouse model.

Gal3 is proposed to be a biomarker for infectious disease (37). While the serum level of gal3 is low in healthy individuals, it is significantly elevated in patients with candidiasis compared to those with non-infectious inflammation (37). It appears that gal3 remains largely intracellular in healthy homeostatic condition and is released into circulation in systemic *Candida* infection. Since recombinant gal3 enhances human neutrophil phagocytosis of *Candida parapsilosis* yeasts and *C. albicans* hyphae (14), released gal3 in the body may enhance uptake of *Candida* by neutrophils. Here, we addressed cytosolic gal3 in anti-*Candida* functions and found that both human and mouse neutrophils constitutively express cytosolic gal3 and it is redistributed and becomes detectable on the surface after stimulation. Thus it seems unlikely that gal3 participates in neutrophil recognition of *Candida*. Employing β-lactose (up to 50 mM), a cell-impermeable pan-galectin inhibitor, we observed that neutrophil ROS production is not affected by the inhibitor after stimulation by opsonized *Candida* (unpublished data). Treatment with TD139, a cell-permeable gal3 inhibitor (F.-T. L., unpublished observation), however, increases neutrophil ROS production. Thus, it is cytosolic gal3 that negatively regulates neutrophil ROS production. Since neutrophils are of primary importance in defense against systemic candidiasis, the negative role of cytosolic gal3 in neutrophil anti-*Candida* functions cannot be ignored.

Cytosolic gal3 has been shown to bind to Alix, a subunit of the endosomal sorting complex, thereby promoting intracellular trafficking and surface expression of epidermal growth factor (EGF)-receptor in keratinocytes and enhancing EGF-induced migration (7). Gal3 is also known to serve as a binding partner of activated K-Ras, through which it enhances PI3K and Raf-1 activation (10). Gal3 binding to Bax protein in human thyroid carcinoma cells responding to apoptosis stimulus interferes with apoptosis (12). We show that cytosolic gal3 directly associates with Syk upon CR3 engagement. We reason that gal3 inhibits Syk phosphorylation through this interaction, thereby regulating a CR3 downstream signaling pathway. Syk being downstream of integrin engagement can be negatively regulated by E3 ligase Cbl resulting in degradation or by SH2 domain tyrosine phosphatase SHP-1 (38). Our data show that the levels of Syk protein in *gal3^+/+^* and *gal3^−/−^* neutrophils remain unchanged after stimulation, suggesting that Cbl is not involved in gal3-mediated inhibition of Syk activation. It still awaits to be clarified whether gal3 regulates Syk through recruitment of SHP-1 or through other mechanisms.

Gal3 has been shown to modulate immune response to infections in different animal models. In pulmonary *Streptococcus pneumonia* infection, the number of infiltrating neutrophils in the lungs of *gal3^−/−^* is lower than in *gal3^+/+^* mice (33). In experimental multiple sclerosis mouse model, g*al3^−/−^* mice infected with Theiler’s murine encephalomyelitis virus have reduced CCL2, CCL5, CCL8, and CXCL10 expression and lower number of infiltrating cells in the brain subventricular zone (32). Infected with *Trypanosoma cruzi, gal3^−/−^* mice exhibit higher levels of parasitemia but have reduced cellular infiltration and tissue damage in the heart (39). Systemic infection by *C. albicans* results in inflammation-induced renal pathology leading to death while *Ccr1^−/−^* or *Ifnar1^−/−^* mice have reduced number of renal infiltrating cells and develop less severe renal pathology and better survival (40, 41), suggesting that neutrophil infiltration in systemic candidiasis denotes poor prognosis. Although studies of bacterial and parasitic infections demonstrate that the presence of gal3 affects neutrophil infiltration, we observed in this study that while the expression of neutrophil-attracting chemokines and renal neutrophil infiltration are comparable in *gal3^+/+^* and *gal3^−/−^* mice after systemic *Candida* infection, *gal3^−/−^* mice have less renal pathology and better survival than *gal3^+/+^* mice. Renal infiltrating neutrophils from *gal3^−/−^* mice produce higher level of ROS and kill *Candida* more efficiently. Thus, gal3 functions to inhibit neutrophil ROS production and the ability of neutrophils to kill *Candida* in systemic candidiasis rather than positively modulate cellular infiltration like that in bacterial and parasitic infections.

Innate cells other than neutrophils are also known to be involved in protecting the host from *Candida* infection. Depleting monocyte/macrophage by clodronate-containing liposomes abolishes host resistance to systemic candidiasis (42). Depleting CCR2^+^ cells interrupts monocyte infiltration and increases fungal burdens in kidney, brain, and spleen during early phase of *Candida* infection (43). Reduced number of renal infiltrating macrophages in infected CX_3_CR1-deficient mice significantly exacerbates systemic candidiasis (34). Moreover, E3 ubiquitin ligase CBLB protein negatively regulates macrophage and dendritic cell anti-*Candida* functions with *Cblb^−/−^* mice more resistant to *Candida* infection (44). Knocking out Syk in dendritic cells with CD11c promoter-driven Syk-deletion demonstrates that dendritic cells contribute to clearance of *Candida* through production of IL-23 which is important to NK cell secretion of GM-CSF (45). NK cell-derived GM-CSF promotes neutrophil ROS production and fungicidal activity (46). We discovered that in addition to regulation of neutrophil ROS production, endogenous gal3 was also involved in macrophage activation, monocyte, and dendritic cell recruitment to kidneys in systemic candidiasis. Consistent with previous findings (47, 48), we found that gal3 plays a positive role in activation of M2 macrophage marked by lower MHC II and higher CD206 expression. Moreover, the negative effect of gal3 is abolished by depleting either neutrophils or macrophages (Figures S6A,B in Supplementary Material). These results show that gal3 is involved not only in neutrophil but also other myeloid cell anti-*Candida* immune response. While we delineated the mechanism of how gal3 regulates neutrophil ROS production through suppression of Syk activation, the mechanism of how gal3 regulates macrophages and dendritic cells in systemic candidiasis warrants further study.

While our study reveals the negative role of gal3 in systemic infection with *Candida* (5 × 10^5^ CFU), Linden et al. reported an opposite effect (49). In their study, *gal3^+/+^* mice infected with low lethal dose of *Candida* (1.0 × 10^5^ CFU) have fewer abscesses in the kidneys, lower fungal burdens and better survival than *gal3^−/−^* mice. Exaggerated inflammatory infiltration and uncontrolled inflammatory responses were attributed to higher mortality in *gal3^−/−^* mice. Previous study employing *Ifnar1^−/−^* mice showed opposite effects of type I interferon in host responses to high lethal (5.0 × 10^5^ CFU), low lethal (1.0 × 10^5^ CFU), and sublethal (5.0 × 10^4^ CFU) doses of *Candida* challenge (41). We speculate that different sizes of inoculum, different commensal flora in the mice resulting from different housing conditions, as well as the difference in the embryonic stem cells (WW6 ES cells vs. D3 ES cells) employed and the backcross strategy may account for our opposite observations. However, our adoptive transfer experiments showing that transferred *gal3^−/−^* neutrophils clear *Candida* more efficiently in recipient mice than *gal3^+/+^* neutrophils support our conclusion that cell intrinsic gal3 negatively regulates host defense against systemic candidiasis.

Our results show that gal3 regulates both human and mouse neutrophil ROS production and that the efficiency of neutrophils to kill *Candida* varies depending on the isolates they encountered. Gal3 deficiency enhances the ability of mouse neutrophils to kill when encountering “hard-to-kill” *Candida*. Based on galectin CRD binding to β-galactoside, Nilsson and colleagues developed a novel gal3 antagonist, TD139 (50). While TD139 binds to gal1, 3 and 7, its binding affinity to gal3 is much higher than binding to gal7 and gal1 (27). Since TD139 can get inside the cell, our results showing this compound inhibits neutrophil ROS production suggest that TD139 functions intracellularly to interfere gal3 and Syk association. Treatment with TD139 has been reported to reduce disease severity in animal models of idiopathic pulmonary fibrosis (51), Concanavalin A-induced hepatitis (52), and type 1 diabetes (53). These studies demonstrated a potential treatment strategy for using TD139 in diseases where gal3 contributes positively to pathogenesis. Phase I clinical trial has demonstrated that TD139 is safe and tolerable when used in healthy volunteers (http://ClinicalTrials.gov identifier, NCT02257177). Thus, using TD139 to block the activity of gal3 is a viable option for enhancing neutrophil killing of *Candida* possibly when used in combination with antifungal drug treatment.

In summary, this study shows that the dynamics of gal3 in cytosol changes after neutrophils encountering *Candida*. Gal3 which physically interacts with Syk negatively affects CR3 downstream Syk phosphorylation and thus negatively regulates Syk-mediated ROS production. We also demonstrate in adoptive transfer experiments that cell intrinsic gal3 in neutrophils dampens host defense against candidiasis. The effect of gal3 in neutrophil interaction with *C. albicans* is generalizable to “hard-to-kill” clinical isolates. This study uncovers the new role of cytosolic gal3 in binding to Syk and in modulating CR3 downstream signal pathway in response to *Candida*. Importantly, treatment with antagonist TD139 and silencing gal3 expression in human neutrophils enhances neutrophil anti-*Candida* function. Our study points to the possibility of targeting gal3 as a potential therapeutic strategy for controlling systemic candidiasis.

## Accession Numbers

The accession numbers in the UniPortKB/SwissProt database of the proteins mentioned in this study are follows: mouse gal3, P16110; human gal3, P17931; CD11b, P05555; Syk, P43404; and NCF-1, Q09014.

## Author Contributions

S-YW contributed to the conception and design of the work; devoted to the acquisition, analysis, and interpretation of data for the work; drafted the work; and revised the manuscript critically for important intellectual content. J-HH and W-YC devoted to the acquisition, analysis, and interpretation of data for the work and drafted the work. Y-C Chen, Y-C Chan, and C-HL contributed to the conception and design of the work and provided reagents that are required resolving questions related to the integrity of the work. F-TL contributed to the conception and design of the work, provided reagents, and revised the manuscript critically for important intellectual content. BW-H contributed to the conception and design of the work, devoted to the interpretation of data for the work, drafted the work, revised the manuscript critically for important intellectual content, final approval of the version to be published, and agreed to be accountable for all aspects of the work in ensuring that questions related to the accuracy or integrity of any part of the work are appropriately investigated and resolved.

## Conflict of Interest Statement

The authors declare that the research was conducted in the absence of any commercial or financial relationships that could be construed as a potential conflict of interest.

## References

[1] Dumic J Dabelic S Flogel M. Galectin-3: an open-ended story. Biochim Biophys Acta (2006) 1760(4):616–35. 10.1016/j.bbagen.2005.12.020 16478649

[2] Rabinovich GA Toscano MA. Turning ‘sweet’ on immunity: galectin-glycan interactions in immune tolerance and inflammation. Nat Rev Immunol (2009) 9(5):338–52. 10.1038/nri2536 19365409

[3] Sundblad V Croci DO Rabinovich GA. Regulated expression of galectin-3, a multifunctional glycan-binding protein, in haematopoietic and non-haematopoietic tissues. Histol Histopathol (2011) 26(2):247–65. 10.14670/HH-26.247 21154238

[4] Alves CM Silva DA Azzolini AE Marzocchi-Machado CM Carvalho JV Pajuaba AC Galectin-3 plays a modulatory role in the life span and activation of murine neutrophils during early *Toxoplasma gondii* infection. Immunobiology (2010) 215(6):475–85. 10.1016/j.imbio.2009.08.001 19720428

[5] Chen HY Fermin A Vardhana S Weng IC Lo KF Chang EY Galectin-3 negatively regulates TCR-mediated CD4+ T-cell activation at the immunological synapse. Proc Natl Acad Sci U S A (2009) 106(34):14496–501. 10.1073/pnas.0903497106 19706535 PMC2732795

[6] Ferraz LC Bernardes ES Oliveira AF Ruas LP Fermino ML Soares SG Lack of galectin-3 alters the balance of innate immune cytokines and confers resistance to *Rhodococcus equi* infection. Eur J Immunol (2008) 38(10):2762–75. 10.1002/eji.200737986 18825751

[7] Liu W Hsu DK Chen HY Yang RY Carraway KL III Isseroff RR Galectin-3 regulates intracellular trafficking of EGFR through Alix and promotes keratinocyte migration. J Invest Dermatol (2012) 132(12):2828–37. 10.1038/jid.2012.211 22785133 PMC3496033

[8] Nakahara S Oka N Raz A. On the role of galectin-3 in cancer apoptosis. Apoptosis (2005) 10(2):267–75. 10.1007/s10495-005-0801-y 15843888

[9] Esteban A Popp MW Vyas VK Strijbis K Ploegh HL Fink GR. Fungal recognition is mediated by the association of dectin-1 and galectin-3 in macrophages. Proc Natl Acad Sci U S A (2011) 108(34):14270–5. 10.1073/pnas.1111415108 21825168 PMC3161568

[10] Elad-Sfadia G Haklai R Balan E Kloog Y. Galectin-3 augments K-Ras activation and triggers a Ras signal that attenuates ERK but not phosphoinositide 3-kinase activity. J Biol Chem (2004) 279(33):34922–30. 10.1074/jbc.M312697200 15205467

[11] Li Y Liu L Niu Y Feng J Sun Y Kong X Modified apple polysaccharide prevents against tumorigenesis in a mouse model of colitis-associated colon cancer: role of galectin-3 and apoptosis in cancer prevention. Eur J Nutr (2012) 51(1):107–17. 10.1007/s00394-011-0194-3 21516492

[12] Harazono Y Kho DH Balan V Nakajima K Zhang T Hogan V Galectin-3 leads to attenuation of apoptosis through Bax heterodimerization in human thyroid carcinoma cells. Oncotarget (2014) 5(20):9992–10001. 10.18632/oncotarget.2486 25393982 PMC4259453

[13] Farnworth SL Henderson NC Mackinnon AC Atkinson KM Wilkinson T Dhaliwal K Galectin-3 reduces the severity of pneumococcal pneumonia by augmenting neutrophil function. Am J Pathol (2008) 172(2):395–405. 10.2353/ajpath.2008.070870 18202191 PMC2312371

[14] Linden JR Kunkel D Laforce-Nesbitt SS Bliss JM. The role of galectin-3 in phagocytosis of *Candida albicans* and *Candida parapsilosis* by human neutrophils. Cell Microbiol (2013) 15(7):1127–42. 10.1111/cmi.12103 23279221 PMC3640745

[15] Nieminen J St-Pierre C Sato S. Galectin-3 interacts with naive and primed neutrophils, inducing innate immune responses. J Leukoc Biol (2005) 78(5):1127–35. 10.1189/jlb.1204702 16260586

[16] Gow NA van de Veerdonk FL Brown AJ Netea MG *Candida albicans* morphogenesis and host defence: discriminating invasion from colonization. Nat Rev Microbiol (2012) 10(2):112–22. 10.1038/nrmicro2711 PMC3624162 22158429

[17] Antachopoulos C Walsh TJ Roilides E. Fungal infections in primary immunodeficiencies. Eur J Pediatr (2007) 166(11):1099–117. 10.1007/s00431-007-0527-7 17551753

[18] Horn DL Neofytos D Anaissie EJ Fishman JA Steinbach WJ Olyaei AJ Epidemiology and outcomes of candidemia in 2019 patients: data from the prospective antifungal therapy alliance registry. Clin Infect Dis (2009) 48(12):1695–703. 10.1086/599039 19441981

[19] Lionakis MS Netea MG *Candida* and host determinants of susceptibility to invasive candidiasis. PLoS Pathog (2013) 9(1):e1003079 10.1371/journal.ppat.1003079 23300452 PMC3536687

[20] Kullberg BJ Arendrup MC Invasive candidiasis. N Engl J Med (2015) 373(15):1445–56. 10.1056/NEJMra1315399 26444731

[21] Chen PY Chuang YC Wang JT Sheng WH Yu CJ Chu CC Comparison of epidemiology and treatment outcome of patients with candidemia at a teaching hospital in Northern Taiwan, in 2002 and 2010. J Microbiol Immunol Infect (2014) 47(2):95–103. 10.1016/j.jmii.2012.08.025 23063082

[22] Lionakis MS Lim JK Lee CC Murphy PM. Organ-specific innate immune responses in a mouse model of invasive candidiasis. J Innate Immun (2011) 3(2):180–99. 10.1159/000321157 21063074 PMC3072204

[23] Aratani Y Kura F Watanabe H Akagawa H Takano Y Suzuki K Critical role of myeloperoxidase and nicotinamide adenine dinucleotide phosphate-oxidase in high-burden systemic infection of mice with *Candida albicans*. J Infect Dis (2002) 185(12):1833–7. 10.1086/340635 12085336

[24] Koh AY Kohler JR Coggshall KT Van Rooijen N Pier GB. Mucosal damage and neutropenia are required for *Candida albicans* dissemination. PLoS Pathog (2008) 4(2):e35. 10.1371/journal.ppat.0040035 18282097 PMC2242836

[25] Chao CC Hsu PC Jen CF Chen IH Wang CH Chan HC Zebrafish as a model host for *Candida albicans* infection. Infect Immun (2010) 78(6):2512–21. 10.1128/IAI.01293-09 20308295 PMC2876552

[26] Maqbool M Vidyadaran S George E Ramasamy R. Optimisation of laboratory procedures for isolating human peripheral blood derived neutrophils. Med J Malaysia (2011) 66(4):296–9. 22299545

[27] Hsieh TJ Lin HY Tu Z Lin TC Wu SC Tseng YY Dual thio-digalactoside-binding modes of human galectins as the structural basis for the design of potent and selective inhibitors. Sci Rep (2016) 6:29457. 10.1038/srep29457 27416897 PMC4945863

[28] Vonk AG Netea MG Kullberg BJ. Phagocytosis and intracellular killing of *Candida albicans* by murine polymorphonuclear neutrophils. Methods Mol Biol (2012) 845:277–87. 10.1007/978-1-61779-539-8_18 22328381

[29] Novak R Dabelic S Dumic J. Galectin-1 and galectin-3 expression profiles in classically and alternatively activated human macrophages. Biochim Biophys Acta (2012) 1820(9):1383–90. 10.1016/j.bbagen.2011.11.014 22155450

[30] Shan M Gentile M Yeiser JR Walland AC Bornstein VU Chen K Mucus enhances gut homeostasis and oral tolerance by delivering immunoregulatory signals. Science (2013) 342(6157):447–53. 10.1126/science.1237910 24072822 PMC4005805

[31] Gazendam RP van Hamme JL Tool AT van Houdt M Verkuijlen PJ Herbst M Two independent killing mechanisms of *Candida albicans* by human neutrophils: evidence from innate immunity defects. Blood (2014) 124(4):590–7. 10.1182/blood-2014-01-551473 24948657

[32] James RE Hillis J Adorjan I Gration B Mundim MV Iqbal AJ Loss of galectin-3 decreases the number of immune cells in the subventricular zone and restores proliferation in a viral model of multiple sclerosis. Glia (2016) 64(1):105–21. 10.1002/glia.22906 26337870 PMC4988318

[33] Nieminen J St-Pierre C Bhaumik P Poirier F Sato S. Role of galectin-3 in leukocyte recruitment in a murine model of lung infection by *Streptococcus pneumoniae*. J Immunol (2008) 180(4):2466–73. 10.4049/jimmunol.180.4.2466 18250456

[34] Lionakis MS Swamydas M Fischer BG Plantinga TS Johnson MD Jaeger M CX3CR1-dependent renal macrophage survival promotes *Candida* control and host survival. J Clin Invest (2013) 123(12):5035–51. 10.1172/JCI71307 24177428 PMC3859390

[35] Chen Y Mendoza S Davis-Gorman G Cohen Z Gonzales R Tuttle H Neutrophil activation by murine retroviral infection during chronic ethanol consumption. Alcohol Alcohol (2003) 38(2):109–14. 10.1093/alcalc/agg049 12634256

[36] Bain JM Louw J Lewis LE Okai B Walls CA Ballou ER *Candida albicans* hypha formation and mannan masking of beta-glucan inhibit macrophage phagosome maturation. MBio (2014) 5(6):e01874 10.1128/mBio.01874-14 25467440 PMC4324242

[37] ten Oever J Giamarellos-Bourboulis EJ van de Veerdonk FL Stelma FF Simon A Janssen M Circulating galectin-3 in infections and non-infectious inflammatory diseases. Eur J Clin Microbiol Infect Dis (2013) 32(12):1605–10. 10.1007/s10096-013-1919-4 23828453

[38] Mocsai A Ruland J Tybulewicz VL. The SYK tyrosine kinase: a crucial player in diverse biological functions. Nat Rev Immunol (2010) 10(6):387–402. 10.1038/nri2765 20467426 PMC4782221

[39] Pineda MA Cuervo H Fresno M Soto M Bonay P. Lack of galectin-3 prevents cardiac fibrosis and effective immune responses in a murine model of *Trypanosoma cruzi* infection. J Infect Dis (2015) 212(7):1160–71. 10.1093/infdis/jiv185 25805753

[40] Lionakis MS Fischer BG Lim JK Swamydas M Wan W Richard Lee CC Chemokine receptor Ccr1 drives neutrophil-mediated kidney immunopathology and mortality in invasive candidiasis. PLoS Pathog (2012) 8(8):e1002865. 10.1371/journal.ppat.1002865 22916017 PMC3420964

[41] Majer O Bourgeois C Zwolanek F Lassnig C Kerjaschki D Mack M Type I interferons promote fatal immunopathology by regulating inflammatory monocytes and neutrophils during *Candida* infections. PLoS Pathog (2012) 8(7):e1002811. 10.1371/journal.ppat.1002811 22911155 PMC3406095

[42] Qian Q Jutila MA Van Rooijen N Cutler JE. Elimination of mouse splenic macrophages correlates with increased susceptibility to experimental disseminated candidiasis. J Immunol (1994) 152(10):5000–8. 8176217

[43] Ngo LY Kasahara S Kumasaka DK Knoblaugh SE Jhingran A Hohl TM. Inflammatory monocytes mediate early and organ-specific innate defense during systemic candidiasis. J Infect Dis (2014) 209(1):109–19. 10.1093/infdis/jit413 23922372 PMC3864383

[44] Wirnsberger G Zwolanek F Asaoka T Kozieradzki I Tortola L Wimmer RA Inhibition of CBLB protects from lethal *Candida albicans* sepsis. Nat Med (2016) 22(8):915–23. 10.1038/nm.4134 27428901 PMC6209141

[45] Whitney PG Bar E Osorio F Rogers NC Schraml BU Deddouche S Syk signaling in dendritic cells orchestrates innate resistance to systemic fungal infection. PLoS Pathog (2014) 10(7):e1004276. 10.1371/journal.ppat.1004276 25033445 PMC4102599

[46] Bar E Whitney PG Moor K Reis e Sousa C LeibundGut-Landmann S. IL-17 regulates systemic fungal immunity by controlling the functional competence of NK cells. Immunity (2014) 40(1):117–27. 10.1016/j.immuni.2013.12.002 24412614

[47] MacKinnon AC Farnworth SL Hodkinson PS Henderson NC Atkinson KM Leffler H Regulation of alternative macrophage activation by galectin-3. J Immunol (2008) 180(4):2650–8. 10.4049/jimmunol.180.4.2650 18250477

[48] Corna G Campana L Pignatti E Castiglioni A Tagliafico E Bosurgi L Polarization dictates iron handling by inflammatory and alternatively activated macrophages. Haematologica (2010) 95(11):1814–22. 10.3324/haematol.2010.023879 20511666 PMC2966902

[49] Linden JR De Paepe ME Laforce-Nesbitt SS Bliss JM. Galectin-3 plays an important role in protection against disseminated candidiasis. Med Mycol (2013) 51(6):641–51. 10.3109/13693786.2013.770607 23488971 PMC3713172

[50] Nilsson U Leffler H Mukhopadhyay B Rajput V, Inventor; Galecto Biotech Ab, Assignee. Galectoside Inhibitors of Galectins. US patent 9353141 (2016).

[51] Mackinnon AC Gibbons MA Farnworth SL Leffler H Nilsson UJ Delaine T Regulation of transforming growth factor-beta1-driven lung fibrosis by galectin-3. Am J Respir Crit Care Med (2012) 185(5):537–46. 10.1164/rccm.201106-0965OC 22095546 PMC3410728

[52] Volarevic V Milovanovic M Ljujic B Pejnovic N Arsenijevic N Nilsson U Galectin-3 deficiency prevents concanavalin A-induced hepatitis in mice. Hepatology (2012) 55(6):1954–64. 10.1002/hep.25542 22213244

[53] Saksida T Nikolic I Vujicic M Nilsson UJ Leffler H Lukic ML Galectin-3 deficiency protects pancreatic islet cells from cytokine-triggered apoptosis in vitro. J Cell Physiol (2013) 228(7):1568–76. 10.1002/jcp.24318 23280610

