# Supplementary material for: Cell Intrinsic Galectin-3 Attenuates Neutrophil ROS-Dependent Killing of Candida by Modulating CR3 Downstream Syk Activation
Source: Front Immunol. 2017 Feb 3;8:48. doi: 10.3389/fimmu.2017.00048 (PMC5289966; doi:10.3389/fimmu.2017.00048)
Supplement: Supplementary file 2 [file Data_Sheet_1.DOCX]

**Cell Intrinsic Galectin-3 Attenuates Neutrophil ROS-Dependent Killing of *Candida* by Modulating CR3 Downstream Syk Activation**

Sheng-Yang Wu, Juin-Hua Huang, Wen-Yu Chen, Yi-Chen Chan, Chun-Hung Lin, Yee-Chun Chen, Fu-Tong Liu, and Betty A. Wu-Hsieh^*^

*Correspondence: bwh@ntu.edu.tw


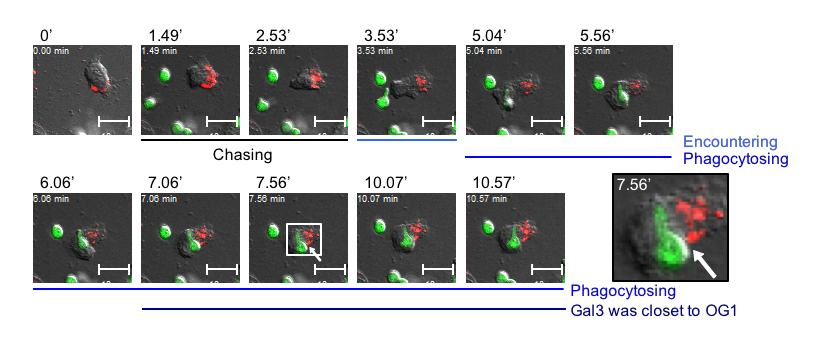


**Supplementary Figure 1. Cytosolic gal3 does not directly interact with engulfed *Candida*.** Bone marrow neutrophils were stimulated with opsonized *Candida* GFP^+^ OG1. Time-lapse fluorescence microscopy and automated imaging were used to observe interaction between intracellular gal3 and *Candida*. Anti-gal3 antibody (red) was delivered to neutrophil intracellularly by PULSin reagent. Arrow indicates where gal3 was closest to *Candida*. See also Movie S1.


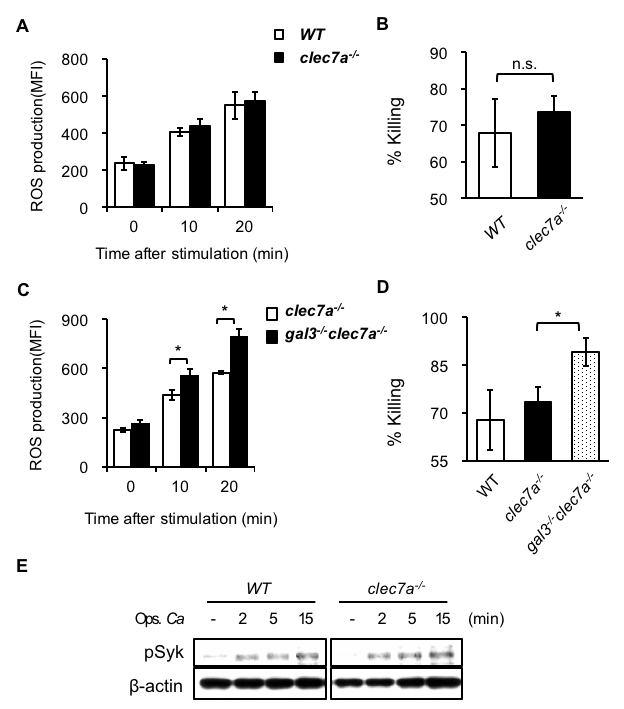


**Supplementary Figure 2. Dectin-1 is not involved in Syk-mediated neutrophil anti-*Candida* function.** WT, *clec7a^-/-^* and *gal3^-/-^clec7a^-/-^* neutrophils were stimulated with opsonized *Candida* (Ops. *Ca*). (**A, C**) Bone marrow neutrophils were pre-stained with CM-H_2_DCFDA before addition of opsonized *Candida* at MOI = 0.5. The level of ROS production is shown as MFI. (**B, D**) % Killing of fungus was determined. n = 3. Data are presented as mean ± SD. n.s., not significant, as analyzed by Mann-Whitney test. (**E**) Cell lysates were collected at different time points after stimulation and subjected to Western blot analysis with anti-p-Syk antibody. β-actin was used as loading control.


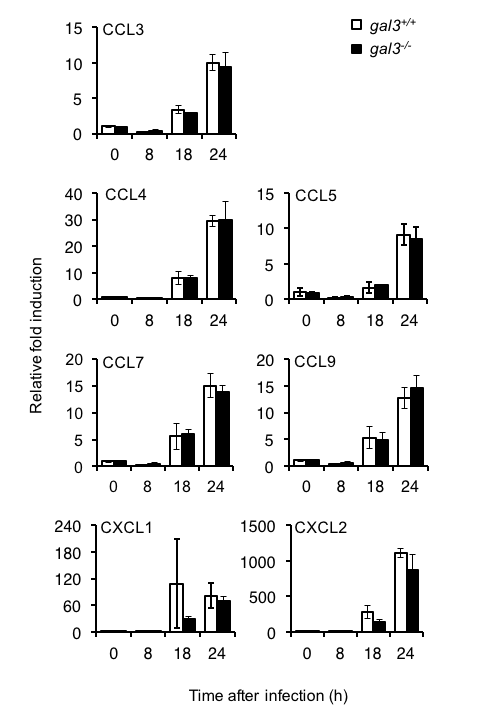
**Supplementary Figure 3. Gal3 does not affect neutrophil-attracting chemokine expression after *Candida* infection.** *Gal3*^+/+^ and *gal3*^-/-^ mice were infected intravenously with 5×10^5^ of *Candida*. Kidneys were collected from uninfected and infected mice at different time points after infection. The relative levels of chemokine mRNA expression were quantified by RT-qPCR. n = 3. Data are presented as mean ± SD. *, *p* < 0.05, as analyzed by Mann-Whitney test.


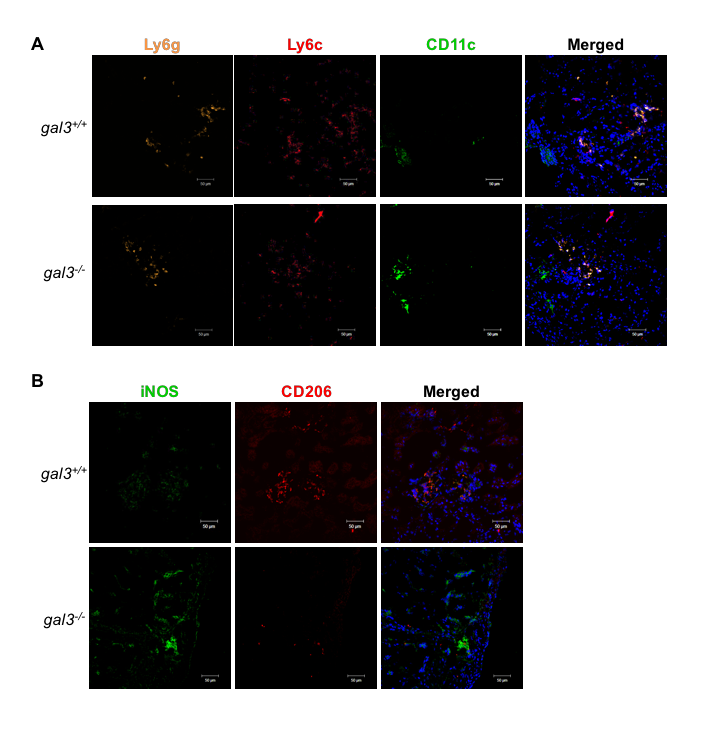


**Supplementary Figure 4. Renal infiltrating cell populations in infected *gal3^+/+^* and *gal3^-/-^* mice.** *Gal3*^+/+^ and *gal3*^-/-^ mice were infected intravenously with 5×10^5^ of *Candida*. Kidneys were collected from uninfected and infected mice at 18 h after infection. Frozen kidneys were embedded in OCT and subject to cryosectioning. Sections were stained Ly6g (Neutrophil marker), Ly6c (monocyte marker), DC11c (DC marker) (**A**), and with anti-iNOS (M1 maker), CD206 (M2 marker) (**B**) antibodies.


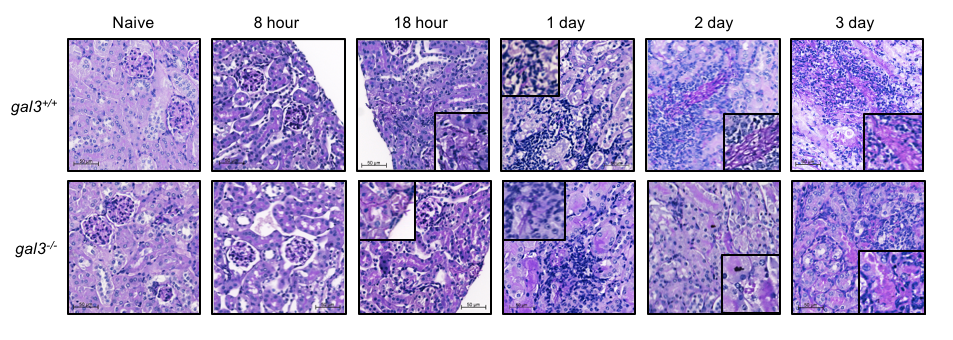
**Supplementary Figure 5. Hyphal formation and growth of *Candida* in kidneys from *gal3^+/+^* and *gal3^-/-^* mice.** *Gal3^+/+^* and *gal3^-/-^* mice were infected intravenously with 5×10^5^ of *Candida*. Kidneys were fixed in formalin before PAS stain. n = 3. The magnification is 200× and that of the inset is 400×.

**Supplementary Figure 6. Depletion of neutrophil or monocyte/macrophage abolishes the negative effect of gal3 on survival after *Candida* infection.**
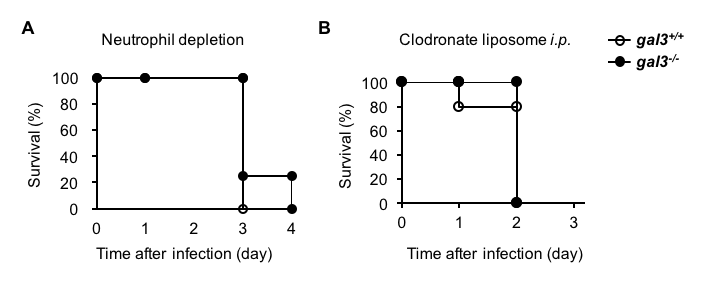
Mice were given intraperitoneal injection of 500 μg of anti-Ly6G antibody (clone 1A8) at the time of infection (**A**) or 200 μl of clodronate-containing liposome (clophosome) 1 day before infection (**B**). The survival of infected mice was followed until day 3-4 after infection. Data were analyzed by log-rank test. **A**, n = 4; **B**, n = 5.

**Table S1. Antibodies used in surface and intracellular staining**

| **Antibody** | **Clone** | **Species** | **Conjugation** | **Company** |
| --- | --- | --- | --- | --- |
| Anti-gal3 | M3/38 | Rat IgG | Unconjugated | BioLegend |
| Anti-rat IgG | Polyclone | Goat IgG | PE | eBioscience |
| Anti-rat IgG | Polyclone | Goat IgG | Alexa 488 | Jackson Immuno Research |
| Anti-CD45 | 30-F11 | Rat IgG | PE-Cy7 | BioLegend |
| Anti-Ly6G | 1A8 | Rat IgG | APC | BioLegend |
| Anti-Gr-1 | RB6-8C5 | Rat IgG | FITC | BioLegend |
| Anti-CD11b | M1/70 | Rat IgG | PE | eBioscience |
| Anti-F4/80 | BM8 | Rat IgG | APC | BioLegend |
| Anti-MHC II | M5/114.15.2 | Rat IgG | PE, FITC | eBioscience |

**Table S2. Antibodies used in immunofluorescence staining**

| **Antibody** | **Clone** | **Species** | **Conjugation** | **Company** |
| --- | --- | --- | --- | --- |
| Anti-gal3 | M3/38 | Rat IgG | Unconjugated | BioLegend |
| Anti-Syk | Polyclone | Rabbit IgG | Unconjugated | GeneTex |
| Anti-PKCβ2 | Polyclone | Rabbit IgG | Unconjugated | Santa Cruz |
| Anti-CD11b | M1/70 | Rat IgG | APC | eBioscience |
| Anti-F4/80 | BM8 | Rat IgG | APC | BioLegend |
| Anti-Ly6C | HK1.4 | Rat IgG | PE | BioLegend |
| Anti-Ly6G | 1A8 | Rat IgG | APC | BioLegend |
| Anti-CD11c | HL3 | Rat IgG | FITC | BD Pharmingen |
| Anti-CD206 | MR6F3 | Rat IgG | PE | eBioscience |
| Anti-iNOS | Polyclone | Rabbit IgG | Unconjugated | BD Transduction Laboratories |
| Anti-rabbit IgG | Polyclone | Goat IgG | Alexa 488 | Jackson Immuno Research, West Grove, PA, USA |
| Anti-rat IgG | Polyclone | Goat IgG | Alexa 647 | Jackson Immuno Research |

**SUPPLEMENTARY METHODS**

**Mice.** Wild-type, *Itgam*^-/-^ and *ncf-1*^-/-^ mice were originally purchased from the Jackson Laboratories (Bar Harbor, ME, USA). *Gal3^−/−^* mice were generated in the laboratories of Dr. F.-T. Liu (1), and *clec7a*^-/-^ mice from Dr. Gordon D. Brown (2). *Gal3*^-/-^*Itgam*^-/-^ were generated by crossing *gal3*^-/-^ with *Itgam*^-/-^ mice, respectively. Wild-type, *gal3*^-/-^, littermate control of *gal3*^-/-^ and *ncf-1*^-/-^ mice were bred at the National Laboratory Animal Center, Taiwan. *Clec7a*^-/-^, *Itgam*^-/-^, *gal3*^-/-^*Itgam*^-/-^ mice were bred at the Laboratory Animal Center of National Taiwan University College of Medicine. All mice used in this study were on C57BL/6 background and maintained under specific pathogen-free (SPF) conditions. Mice at 6 - 12 weeks of age were used in all of the experiments.

**Isolation and staining cells collected from blood and kidney.** Blood was collected and treated with RBC lysis buffer to obtain peripheral blood leukocytes. To isolate renal infiltrating cells, kidneys were collected after whole body perfusion and cut into small pieces in RPMI 1640 medium, treated with 0.2 mg/ml of Liberase TM (Roche) in 5 ml at 37 °C for 30 min. The suspensions were allowed to pass through 24-gauge needle twice before centrifugation at 300 × g for 5 min. Pellets were resuspended in 7 ml of 45% percoll which was overlaid on 3 ml of 81% percoll. After centrifugation at 1400 × g for 20 min, cells at the interface were collected and stained with antibodies listed in Supplemental Information. The percentage of neutrophils, macrophages, monocytes and dendritic cells in the total leukocyte population was analyzed by flow cytometry.

**Fungicidal activity assay.** The assay was adopted from Method in Molecular Biology (3). *Candida* yeasts were grown in Sabouraud-dextrose broth (pH = 5.6) in 37^o^C with shaking at 150 × g for 16-18 h. Bone marrow neutrophils and CD45^+^Ly6G^+^ renal infiltrating cells were subject to killing assay. Cells were suspended in RPMI complete medium, seeded in 96-well plate at 5 × 10^5^ per well. After 30 min incubation to allow adherence, 1 × 10^4^ opsonized *Candida* yeasts were added to the wells and the plates were centrifuged at 1,000 × g for 3 min. Wells containing 1 × 10^4^ opsonized *Candida* yeasts without neutrophils was used as control. The plates were left in 37^o^C incubator for 20 min. Supernatants in control wells and experimental groups were plated on YPD plate to quantify the numbers of control and uningested yeasts. The number of ingested yeasts (N_0h_) = the number of yeasts in control well – the number of uningested yeasts. The rest of the wells were incubated for another 1.5 h and the cells were lysed by H_2_O (pH = 11) to release intracellular yeasts. The lysates were plated and the number of viable *Candida* yeasts released from cells was determined. % killing = (N_0h_-N_1.5h_)/N_0h_ ×100 %, where N = the number of intracellular *Candida*.

**Western blot analysis.** Neutrophils were stimulated with or without opsonized *Candida* yeasts (MOI = 2). After stimulation, cells were lysed with PhosphoSafe Extraction Reagent (EMD Millipore). Cell lysates were separated by electrophoresis at 10 % SDS-polyacrylamide gel and transferred to Immobilon-P membrane (Millipore). Membrane was blocked with 5% nonfat milk (Fluka) for 1 h and incubated in buffer containing rabbit anti-pSyk (Tyr575), -pPKC (Ser660) (Epitomics), -pAkt (Thr308), -pp38 (Thr180/Tyr182), -p-p40^phox^ (Thr154) (Cell Signaling), and -pPLCγ II (Tyr759), -pPKCα (Thr638), -pPKCβ1 (Thr642), -pPKCγ (Thr674), -pPKCδ (Ser645), -pPKCη (Thr655) (Millipore) or polyclonal goat anti-galectin-3 antibodies at 4 °C overnight. Membrane was then incubated with buffer containing goat anti-rabbit IgG-HRP antibody (GeneTex) or donkey anti-goat IgG-HRP antibody (R&D) for 1 h. Western Chemoluminescent HRP substrate was used as directed (Millipore or GE healthcare). β-actin was used as a loading control.

**Kidney histology.** Mouse was anesthetized and perfused with 20 ml sterile PBS buffer before kidneys were collected. Kidneys were fixed with 10% formalin and embedded in paraffin wax. Tissue sections were subject to H&E and PAS stain and viewed under microscope Axio Scope.A1 (Zeiss).

**Tissue and cell immunofluorescence staining.** Kidneys were embedded in O.C.T. and allowed to freeze in -80 °C overnight. Five µm thick tissue sections were cut and mounted on gelatin-coated slides. Slides were fixed in methanol/acetone (1:1) solution for 20 min before subject to immunofluorescence stating. Bone marrow neutrophils and infiltrating cells isolated from kidney were cytospun onto microscope slides. Either cryosections or cytospun cells were fixed with 3% paraformaldehyde (PFA) and permeabilized with 0.5% Triton X-100. Tissue sections or cells were blocked with PBS containing 5% FBS and stained with primary antibodies overnight at 4 °C. Slides were then stained with secondary antibodies for 45 min. Cell nuclei were stained with Hoechst 33258. The images were viewed under confocal microscope (Zeiss Axiovert 100TV, Carl Zeiss Inc., Jena, Germany) and analyzed by Zen software (Carl Zeiss Inc.) and ImageJ (NIH) software.

**Kinase inhibitors used in experiments.** Bay 61-3606 (Syki), SP600125 (JNKi), U1026 (ERKi), SB203580 (p38i) (all from Calbiochem) or Ro 318220 (PKCi), Rottlerin (PKCδi) (both from Cayman), Ro 6976 (PKCα+β1i), LY 333531 (PKCβi), PKCη pseudopeptide (all from Millipore)

**TD139 treatment.** TD139 powder was reconstituted in 100% DMSO. Human neutrophils were pretreated with 250 μM TD139 in RPMI complete medium for 4 h before stained with CM-H_2_DCFDA for 30 min. At 20 min after stimulation by opsonized *Candida*, ROS production was analyzed by flow cytometry.

**Live cell imaging.** Alexa 647-anti-gal3 antibody (BioLegend) was delivered to neutrophils intracellularly by PULSin reagent (Polyplus-transfection) according to the manufacturer recommendation. Neutrophils were seeded in microscopy chamber (1 μ-Slide 8 well ibiTreat plates, ibidi) and *Candida* OG1 were added to the wells. Intracellular events were monitored by inverted confocal microscope LSM 780 AxioObserver Z1 for three-color and differential intensity contrast imaging.

**Accession numbers.** The accession numbers in the UniPortKB/SwissProt database of the proteins mentioned in this study are follows: Mouse gal3, P16110; Human gal3, P17931; CD11b, P05555; Syk, P43404; NCF-1, Q09014.

**SUPPLEMENTARY REFERENCES**

1. Hsu DK, Yang RY, Pan Z, Yu L, Salomon DR, Fung-Leung WP, et al. Targeted disruption of the galectin-3 gene results in attenuated peritoneal inflammatory responses. *Am J Pathol* (2000) **156**(3):1073-83. doi: 10.1016/S0002-9440(10)64975-9.

2. Taylor PR, Tsoni SV, Willment JA, Dennehy KM, Rosas M, Findon H, et al. Dectin-1 is required for beta-glucan recognition and control of fungal infection. *Nat Immunol* (2007) **8**(1):31-8. doi: 10.1038/ni1408.

3. Vonk AG, Netea MG, Kullberg BJ. Phagocytosis and intracellular killing of Candida albicans by murine polymorphonuclear neutrophils. *Methods Mol Biol* (2012) **845**:277-87. doi: 10.1007/978-1-61779-539-8_18.
